# The impact of muscle relaxation techniques on the quality of life of cancer patients, as measured by the FACT-G questionnaire

**DOI:** 10.1371/journal.pone.0184147

**Published:** 2017-10-19

**Authors:** Paula Parás-Bravo, Paloma Salvadores-Fuentes, Cristina Alonso-Blanco, María Paz-Zulueta, Miguel Santibañez-Margüello, Domingo Palacios-Ceña, Ester Boixadera-Planas, César Fernández-de-las-Peñas

**Affiliations:** 1 Department of Nursing, University of Cantabria, Santander, Spain; 2 Department of Nursing, University Rey Juan Carlos, Alcorcón, Spain; 3 Department Physical Therapy, Occupational Therapy, Rehabilitation, and Physical Medicine, University Rey Juan Carlos, Alcorcón, Spain; 4 Statistical Service of the Universitat Autònoma de Barcelona, Barcelona, Spain; Connecticut Children's Medical Center, UNITED STATES

## Abstract

**Introduction:**

Patients with cancer frequently suffer from emotional distress, characterized by psychological symptoms such as anxiety or depression. The presence of psychological symptoms combined with the complex nature of oncology processes can negatively impact patients’ quality of life. We aimed to determine the impact of a relaxation protocol on improving quality of life in a sample of oncological patients treated in the Spanish National Public Health System.

**Materials and methods:**

We conducted a multicenter interventional study without a control group. In total, 272 patients with different oncologic pathologies and showing symptoms of anxiety were recruited from 10 Spanish public hospitals. The intervention comprised abbreviated progressive muscle relaxation training, according to Bernstein and Borkovec. This was followed by weekly telephone calls to each patient over a 1-month period. We collected sociodemographic variables related to the disease process, including information about mental health and the intervention. Patients’ quality of life was assessed using the Functional Assessment of Cancer Therapy-General (FACT-G) questionnaire. Bivariate and univariate analyses were performed, along with an analysis of multiple correspondences to identify subgroups of patients with similar variations on the FACT-G.

**Results:**

Patients showed statistically significant improvements on the FACT-G overall score (W = 16806; *p*<0.001), with an initial mean score of 55.33±10.42 and a final mean score of 64.49±7.70. We also found significant improvements for all subscales: emotional wellbeing (W = 13118; *p*<0.001), functional wellbeing (W = 16155.5; *p*<0.001), physical wellbeing (W = 8885.5; *p*<0.001), and social and family context (W = −1840; *p* = 0.037).

**Conclusions:**

Patients with cancer who learned and practiced abbreviated progressive muscle relaxation experienced improvement in their perceived quality of life as measured by the FACT-G. Our findings support a previous assumption that complementary techniques (including relaxation techniques) are effective in improving the quality of life of patients with cancer.

## Introduction

The complexity of cancer processes and treatment is associated with emotional distress [1–3]. Consequently, many patients experience symptoms of anxiety and/or depression [4,5]. Approximately one-third of patients with cancer are estimated to suffer from some type of mental disorder during the course of active treatment (20% of patients suffer from anxiety and 13% show signs of depression) [6,7]. Fatigue is also present in 50–90% of patients and pain in 20–50% [8], with pain experienced by 90% of patients when cancer is at advanced stages [9]. In addition, nausea is experienced by around 42% of patients with cancer [10]. These cancer-associated symptoms negatively affect patients’ quality of life; therefore, implementation of interventions to improve these complaints should be a primary objective of health services.

In this context, complementary and alternative medicine (CAM) is gaining increased importance as it contributes to improving the emotional state, fatigue, quality of life, and adherence to treatment in patients who suffer from different types of tumors [11–15]. The National Centre of Complementary and Alternative Medicine notes CAM therapies can be classified into two categories: those related to natural products (e.g., vitamins and minerals) and those involving mind-body practices (e.g., acupuncture, massage, meditation, movement therapy, yoga, hypnotherapy, healing touch, and muscle relaxation) [16].

The physiological foundations of muscle relaxation techniques place the origin of negative emotional states with an excess of neuromuscular tension [17]. By achieving muscle relaxation, relaxation of the mind can be achieved, along with a general state of wellbeing. There is evidence supporting that therapies involving relaxation interventions (i.e., muscle relaxation, acupuncture, yoga, reiki, etc) may improve the quality of life of cancer patients; however, these techniques require several learning sessions, greater follow-up over time, and do not always achieve an increase in quality of life [11,18–25]. These techniques have been used for controlling anxiety in different contexts, including pregnant women [26], fear of dentists [27], pulmonary hypertension [28], and schizophrenia [29]. Improving the quality of life of patients with cancer requires the development of interventions applicable to this unique population. Therefore, this study aimed to determine the impact of a relaxation protocol in improving quality of life in a sample of oncological patients treated in the Spanish National Public Health System.

## Materials and methods

### Design

We used a multicenter pre-post intervention study design without a control group.

### Participants

**Fig 1:**
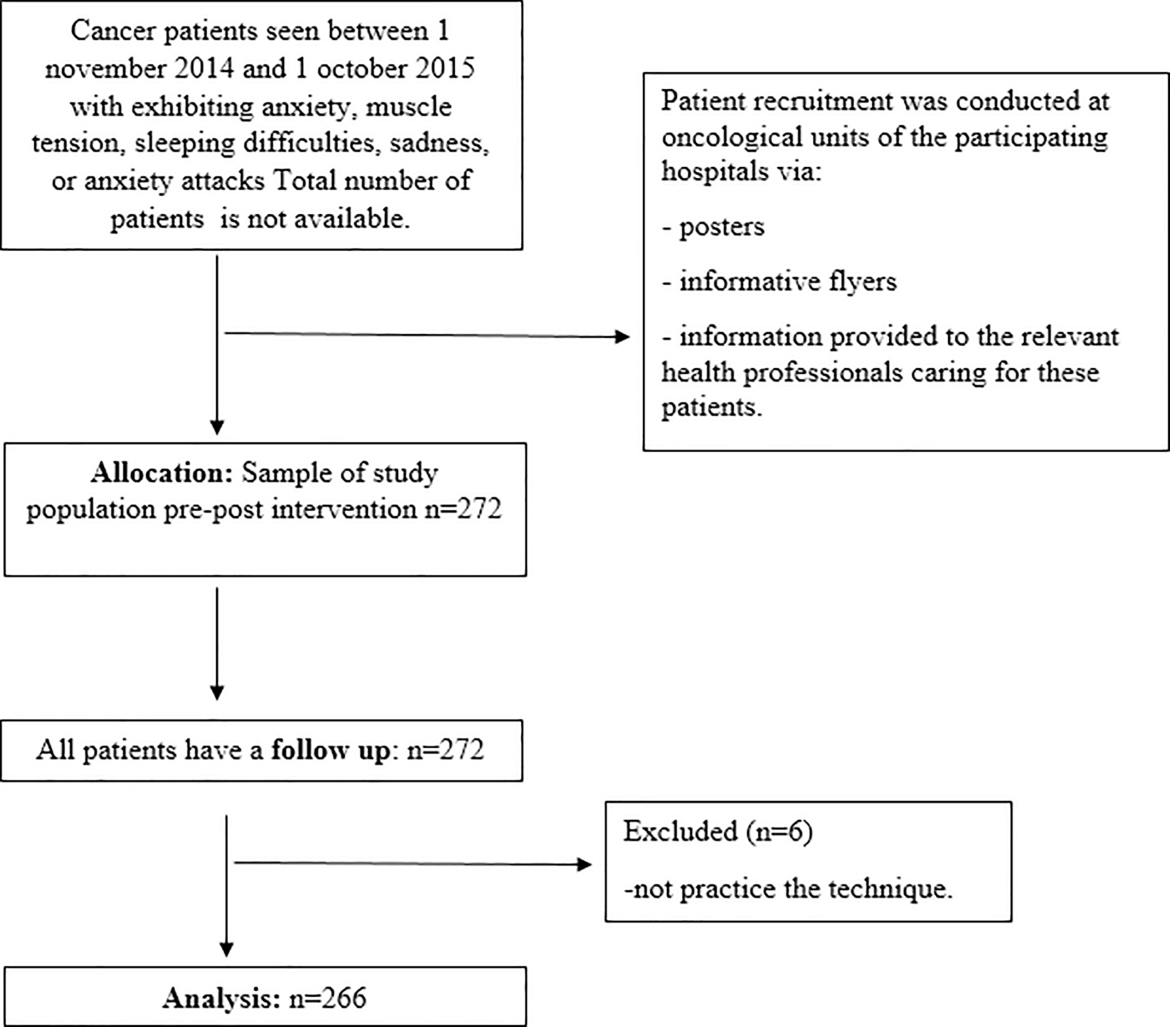
Flowchart of study participants.

This study was performed in the oncological units of 10 hospitals belonging to the Spanish National Public Health System between November 1, 2014 and October 1, 2015. Participation of the hospitals was gradual throughout the study period. In all cases, patient recruitment began after authorization by the ethics committee corresponding to each hospital. The study population included patients with any type of cancer (oncological and/or hematological malignances), of both sexes, older than 18 years, who agreed to participate in the study, and who showed anxiety, muscle tension, sleeping difficulties, sadness, and/or anxiety attacks. Patients were excluded if they showed severe cognitive or physical impairment, were unable to understand or reproduce the relaxation technique used in the intervention, or were in a terminal condition with a prognosis of imminent death. Although no adverse effects have been reported following use of this technique, it is important to highlight that such techniques should not be considered a substitute for medical treatment. Patients suffering from hallucinations, delirium or other psychotic symptoms were also excluded from this study, as the exercises used in the intervention may lead to potentially unpleasant extracorporeal sensations in those patients. Patient recruitment was conducted in the oncology units of participating hospitals via posters, informative flyers, and information provided to the health professionals caring for the patients (i.e., oncologists, nurses, and psychologists). In total, 272 patients from the oncological services of the participating hospitals satisfied all eligibility criteria and agreed to participate. Six patients (2%) did not practice the technique at home, and were excluded from the analysis (Fig 1).

### Data collection

Data were collected using the Functional Assessment of Cancer Therapy-General (FACT-G) questionnaire and an ad hoc data collection notebook. The FACT-G comprises 27 items featuring general questions divided into four quality of life domains: physical wellbeing, social/family wellbeing, emotional wellbeing, and functional wellbeing [30]. Item scores range from 0–4 points. The total score ranges from 0–108 points, with higher scores indicating better quality of life. The FACT-G is considered appropriate for use with patients suffering from any type of cancer [31]. A systematic review found that the FACT-G total and subscale scores had excellent reliability, with Cronbach’s alpha values ranging from 0.71–0.88 [32]. In the present study we used the validated Spanish version of the FACT-G [33].

Information collected in the data collection notebook included: 1) sociodemographic and medical characteristics (medical center, age, gender, marital status, children, and educational level); 2), oncological process (cancer diagnosis, cancer therapy—chemotherapy, radiotherapy, hormone therapy, biological therapy, and surgery—side effects of cancer treatment, cancer pain, and analgesic use); 3) mental health issues (use of anxiolytics/hypnotics/antidepressants, comorbid psychiatric diagnoses, psychiatric-psychological treatment, and use of relaxation techniques); and 4) other variables related to the intervention, such as symptoms that motivated participation in the study and questions including “Have you practiced the technique at home?” and “How many times do you practice the technique in a week?”

This was followed by weekly telephone calls to each patient over a 1-month period.

### Intervention

All participants received an initial guided session to learn abbreviated progressive muscle relaxation training, following Bernstein and Borkovec [34]. This technique consists of contraction and subsequent relaxation of all muscle groups sequentially. In a sitting position and with their eyes closed, participants were instructed to contract and relax the muscles of their hands, forearms, face, neck, shoulders, abdomen, and lower limbs in turn. During implementation of the technique, patients were recommended to perform normal breathing. The initial sessions were conducted individually or in groups, according to the patient’s condition. To unify criteria and reduce possible inter-examiner bias, researchers who conducted the sessions were fully trained regarding the study selection criteria, information provided to participants, data collection procedures, and application of the technique. All researchers were instructed in, and received written guidance about, conducting the relaxation session. The main researcher was present at the first treatment session at all hospital centers to homogenize all aspects of the intervention. A pilot test was performed with the first 30 participating patients. Patients performed the technique in a sitting position, and the sessions were conducted in rooms furnished with armchairs, cushions, pleasant lighting, and a quiet environment. Each session lasted approximately 60 minutes and was divided into four parts: 1) explanation of the characteristics of the abbreviated progressive muscle relaxation training [34]; 2) application of a relaxation session; 3) answering any questions; and 4) data collection using the self-administered FACT-G and data collection notebook.

At the end of the session, patients were provided with information about the intervention, including a brief description of the session based on text and images to support them in performing the technique at their own homes.

### Ethical considerations

This study was approved by the Ethical Committee of Clinical Research accredited by the Ministry of Health, Social Services and Equality (Spain) for each center involved in the study (University Hospital of Getafe, 06/26/2014; Puerta del Hierro-Majadahonda, 07/24/2014; Foundation Alcorcon, 11/03/2014; Fuenlabrada, 12/03/2014; Bellvitge, 09/10/2014; Salamanca, 07/18/2014; Navarra, 03/27/2015; Hospital Germans Trias i Pujol, 11/21/2014; and Cantabria 08/01/2014) [35]. This trial is registered with ISRCTN, under registration number 81335752. Clinical trial registration was delayed as this study was classified by the Spanish Agency of Medicine as an *Observational Study No Epa*, and therefore should not be included in the Spanish Registry of Clinical Studies. In addition, the study sponsor considered this work as a behavioral intervention rather than a clinical trial because of the lack of drugs, biologics, or devices. The authors confirm that there are no ongoing or related trials for this intervention. All procedures were conducted according to the Declaration of Helsinki [36]. All study participants provided written informed consent after they received appropriate information regarding the study aims, potential benefits and possible risks. Data were treated anonymously and confidentially according to the Spanish Personal Data Protection Act [37].

### Sample size calculation

The sample size was calculated using EPIDATA version 4.1. As the inclusion of different hospitals was gradual throughout the study, we estimated the sample size based on an infinite population-based sample. With a 95% confidence level, an expected proportion of 20% of anxiety disorders in the cancer population [6,7], and a maximum error of estimation of 5%, the estimated sample size was 246 patients. An expected loss rate of 5% was assumed; therefore, the final estimated sample size was 259 patients.

### Data analysis

Statistical analyses were performed using SAS version 9.3 (SAS Institute Inc., Cary, NC, USA). The level of statistical significance was set at 0.05. A descriptive univariate analysis to calculate the total score and percentage for each category was performed for categorical variables. Basic descriptive statistics and the Wilcoxon signed-rank test (W) were performed for quantitative variables. For the bivariate analyses, we used the Kruskal-Wallis (KW), chi-square (χ2) or likelihood ratio chi-square (G^2^) (when there are more than 25% of cells with expected counts less than 5) tests for contingency tables, including variables with low expected counts.

We also performed multiple correspondence analyses [38] to detect groups of patients with similar progressions based on FACT-G responses. To perform these analyses, we considered that in each questionnaire item there was a negative, constant, or positive progression according to baseline and final scores. Based on this evolution, we considered the active variables as: GP1, GP6, GS3, GS5, GS6, GS7, GE1, GE2, GE3, GE4, GE5, GF1, GF4, GF6, and GF7 (a minimum of 5% of patients in each type of evolution) (see S1 Table).

From these analyses, we obtained 21 factors which summarized all changes in the sample (see S2 Table). We also performed a classification defining four clusters of patients with similar responses (S1 and S2 Figs).

After obtaining the four patient groups, we analyzed which outcomes (among the various FACT-G items) had a different proportion with regard to the proportion of the sample (hypergeometric distribution). Finally, a bivariate analysis of the variation rate of the FACT-G was performed for each cluster of patients.

## Results

Table 1 describes the sociodemographic and medical characteristics of participating patients for the total sample, and stratified by clusters. No significant differences in sociodemographic features were observed among clusters, except for having children (G^2^ = 89; *p* = 0.031).

**Table 1:** Sociodemographic and medical characteristics of participating patients for the total sample, and stratified by clusters.

CLUSTER												
		TOTAL		cluster 1		cluster 2		cluster 3		cluster 4		*p*
		N	%	N	%	N	%	N	%	N	%	
**MEDICAL CENTER**	** **	** **	** **									
	**University Hospital Marques de Valdecilla, Cantabria.**	16	6.02	10	6.25	2	9.52	2	3.03	2	10.53	
	**Fundación Alcorcón Hospital, Madrid.**	35	13.16	20	12.50	3	14.29	7	10.61	5	26.32	
	**Getafe University Hospital, Madrid.**	17	6.39	14	8.75	1	4.76	—	—	2	10.53	
	**Fuenlabrada University Hospital, Madrid.**	52	19.55	24	15.00	5	23.81	19	28.79	4	21.05	
	**Catalan Institute of Oncology, Hospital University of Bellvitge, Barcelona.**	35	13.16	20	12.50	3	14.29	10	15.15	2	10.53	
	**Catalan Institute of Oncology, Hospital University"Germans Trias i Pujol", Barcelona.**	45	16.92	31	19.38	4	19.05	9	13.64	1	5.26	
	**Sierrallana Hospital, Cantabria.**	4	1.50	3	1.88	—	—	1	1.52	—	—	
	**Hospital of Navarra, Navarra.**	47	17.67	29	18.13	3	14.29	14	21.21	1	5.26	
	**Hospital of Salamanca, Salamanca.**	5	1.88	3	1.88	—	—	1	1.52	1	5.26	
	**Puerta de Hierro-Majadahonda University Hospital, Madrid.**	10	3.76	6	3.75	—	—	3	4.55	1	5.26	
**AGE (years) Mean [SD]**^**a**^	** **	52.56	[11.5]	52.55	[11.57]	50.76	[10.27]	52.36	[10.36]	55.26	[13.85]	0.446*
**GENDER**	** **	** **	** **									
	**Female**	203	76.32	118	73.75	16	76.19	55	83.33	14	73.68	0.460**
	**Male**	63	23.68	42	26.25	5	23.81	11	16.67	5	26.32	
**MARITAL STATUS**	** **	** **	** **									
	**Married**	178	66.92	110	68.75	11	52.38	43	65.15	14	73.68	
	**Single**	39	14.66	22	13.75	5	23.81	12	18.18	—	—	
	**Divorced**	11	4.14	8	5.00	1	4.76	2	3.03	—	—	
	**Widowed**	10	3.76	2	1.25	—	—	3	4.55	5	26.32	
	**Separated**	11	4.14	7	4.38	2	9.52	2	3.03	—	—	
	**Domestic partnership**	17	6.39	11	6.88	2	9.52	4	6.06	—	—	
**CHILDREN**	** **	** **	** **									
	**No**	61	22.93	40	25.00	9	42.86	10	15.15	2	10.53	0.031**
	**Yes**	205	77.07	120	75.00	12	57.14	56	84.85	17	89.47	
**EDUCATIONAL LEVEL**	** **	** **	** **									
	**Elementary**	131	49.25	75	46.88	12	57.14	34	51.52	10	52.63	0.736**
	**Secondary**	87	32.71	59	36.88	5	23.81	18	27.27	5	26.32	
	**University**	48	18.05	26	16.25	4	19.05	14	21.21	4	21.05	
**CANCER DIAGNOSIS**	** **	** **	** **	** **	** **	** **	** **	** **	** **	** **	** **	
	**Lung**	31	11.65	23	14.38	1	4.76	2	3.03	5	26.32	
	**Digestive**	36	13.53	17	10.63	3	14.28	10	15.16	6	31.57	
	**Head and neck**	5	1.88	2	1.26	1	4.76	2	3.04	—	—	
	**Gynecological**	139	52.26	81	50.63	14	66.66	38	57.58	6	31.58	
	**Urinary**	7	2.63	3	1.88	—	—	4	6.06	—	—	
	**Hematological malignancies**	41	15.41	30	18.78	1	4.76	8	12.13	2	10.52	
	**Others**	7	2.63	4	5.51	1	4.76	2	3.04	—	—	
**CANCER THERAPY**	** **	** **	** **									
	**Chemotherapy**	256	96.24	156	97.50	19	90.48	62	93.94	19	100.00	0.218**
	**Radiotherapy**	121	45.49	69	43.13	10	47.62	35	53.03	7	36.84	0.478***
	**Hormone therapy**	52	19.55	28	17.50	7	33.33	17	25.76	—	—	0.006**
	**Biological therapy**	50	18.80	29	18.13	4	19.05	14	21.21	3	15.79	0.938**
	**Surgery to remove cancer**	147	55.26	93	58.13	13	61.90	34	51.52	7	36.84	0.274***
**SIDE EFFECTS OF CANCER TREATMENT**	** **	** **	** **									
	**No**	35	13.16	20	12.50	1	4.76	9	13.64	5	26.32	0.255**
	**Yes**	231	86.84	140	87.50	20	95.24	57	86.36	14	73.68	
**CANCER PAIN**	** **	** **	** **									
	**No**	142	53.38	78	48.75	13	61.90	38	57.58	13	68.42	0.242***
	**Yes**	124	46.62	82	51.25	8	38.10	28	42.42	6	31.58	
**ANALGESICS USE**	** **	** **	** **									
	**No**	—	—	—	—	—	—	—	—	—	—	
	**Yes**	124	100	82	100.00	8	100.00	28	100.00	6	100.00	
**ANXIOLYTICS USE**	** **	** **	** **									
	**No**	182	68.42	109	68.13	13	61.90	47	71.21	13	68.42	0.883
	**Yes**	84	31.58	51	31.88	8	38.10	19	28.79	6	31.58	
**ANTIDEPRESSANTS USE**	** **	** **	** **									
	**No**	234	87.97	142	88.75	18	85.71	60	90.91	14	73.68	0.303**
	**Yes**	32	12.03	18	11.25	3	14.29	6	9.09	5	26.32	
**HYNOTICS USE**	** **	** **	** **									
	**No**	208	78.20	117	73.13	17	80.95	58	87.88	16	84.21	0.073**
	**Yes**	58	21.80	43	26.88	4	19.05	8	12.12	3	15.79	
**PSYCHIATRIC DIAGNOSIS**	** **	** **	** **									
	**No**	259	97.37	154	96.25	21	100.00	65	98.48	19	100.00	
	**Yes**	7	2.63	6	3.75	—	—	1	1.52	—	—	
**PSYCHIATRIC-PSYCHOLOGICAL TREATMENT**	** **	** **	** **									
	**No**	261	98.12	156	97.50	21	100.00	65	98.48	19	100.00	
	**Yes**	5	1.88	4	2.50	—	—	1	1.52	—	—	
**RELAXATION TECHNIQUES**	** **	** **	** **									
	**No**	261	98.12	155	96.88	21	100.00	66	100.00	19	100.00	
	**Yes**	5	1.88	5	3.13	—	—	—	—	—	—	
**STUDY INCLUSION SYMPTOMS**	** **	** **	** **									
	**Anxiety**	261	98.12	159	99.38	21	100.00	65	98.48	16	84.21	0.014**
	**Insomnia**	51	19.17	28	17.50	7	33.33	11	16.67	5	26.32	0.321**
	**Sadness**	15	5.64	9	5.63	1	4.76	4	6.06	1	5.26	0.996**
	**Muscle tension**	3	1.13	1	0.63	1	4.76	—	—	1	5.26	0.183**
**HAVE YOU PRACTICED THE TECHNIQUE AT HOME?**	** **	** **	** **									
	**Week 1**	254	95.49	156	97.50	21	100.00	60	90.91	17	89.47	0.060**
	**Week 2**	257	96.62	156	97.50	21	100.00	62	93.94	18	94.74	0.359**
	**Week 3**	254	95.49	156	97.50	21	100.00	60	90.91	17	89.47	0.060**
	**Week 4**	254	95.49	156	97.50	21	100.00	60	90.91	17	89.47	0.060**
**HOW MANY TIMES HAVE YOU PRACTICED THE TECHNIQUE PER WEEK?**	** **	** **	** **									
	**Week 1 Mean [SD]**	254	6.57 [4.36]	156	6.24 [4.05]	21	5.10 [3.13]	60	8.40 [5.17]	17	6.36 [3.45]	< .001***
	**Week 2 Mean [SD]**	257	6.45 [3.62]	156	6.36 [3.45]	21	5.43 [2.96]	62	7.56 [4.27]	18	4.67 [2.06]	0.002***
	**Week 3 Mean [SD]**	254	6.25 [3.57]	156	6.03 [3.39]	21	5.48 [2.87]	60	7.58 [4.17]	17	4.41 [2.18]	0.004***
** **	**Week 4 Mean [SD]**	254	6.19 [3.63]	156	6.00 [3.45]	21	5.24 [2.96]	60	7.45 [4.32]	17	4.65 [2.42]	0.005***

^a^ SD: Standard deviation

*Kruskal Wallis Test

**LR—Chi-Squared Test

***Chi-Squared Test

More than 90% of patients in each cluster had received chemotherapy, with no significant differences among the clusters (G^2^ = 4.44; *p* = 0.218). However, significant differences were detected in patients undergoing hormone therapy, ranging from 17–33% in clusters 1–3, but none in cluster 4 (G^2^ = 12.42; *p* = 0.006).

Patients in cluster 3 who practiced the technique more times a week on average, exhibited lower levels of pre-session anxiety in all sessions, including the first session (baseline: χ^2^ = 23.15, *p*<0.001; first week: χ^2^ = 18.03, *p*<0.001; second week: χ^2^ = 15.04, *p* = 0.002; third week: χ^2^ = 13.44, *p* = 0.004; fourth week: χ^2^ = 12.92, *p* = 0.005). Table 2 displays the evolution rate of FACT-G overall and subscale scores. There was a significant increase in the mean total score from 55.33±10.42 to 64.49±7.70 (mean change: 9.13±5.38 points, W = 16806; *p*<0.001).

**Table 2:** Variation rates of the FACT-G questionnaire for the total sample.

		N	Mean	SD^a^	*p* *
**TOTAL SCORE**	Baseline	272	55.33	10.42	< .001
	1-month follow-up	266	64.49	7.70	
	Rate of score	266	0.18	0.13	
**PHYSICAL WELL-BEING**	Baseline	272	17.33	4.33	< .001
	1-month follow-up	266	18.71	4.02	
	Rate of score	266	0.08	0.08	
**SOCIAL/FAMILY WELL-BEING**	Baseline	272	18.34	4.38	0.037
	1-month follow-up	266	18.11	3.83	
	Rate of score	266	-0.01	0.11	
**EMOTIONAL WELL-BEING**	Baseline	272	7.64	3.21	< .001
	1-month follow-up	266	11.55	2.21	
	Rate of score	266	0.31	0.31	
**FUNCTIONAL WELL-BEING**	Baseline	272	12.03	5.13	< .001
	1-month follow-up	266	16.12	4.41	
	Rate of score	266	0.28	0.19	

* Wilcoxon Signed Rank Test

^a^SD: Standard deviation

Stratifying scores by subscales showed a significant increase in all subscales: emotional wellbeing (KW = 13118; *p*<0.001); functional wellbeing (KW = 16155.5; *p*<0.001); physical wellbeing (KW = 8885.5; *p*<0.001); and social/family context (KW = −1840; *p* = 0.037). S3 and S4 Figs show the graphical description of the progression of FACT-G scores.

Table 3 shows the rate score of the FACT-G, stratified by subscales and clusters.

**Table 3:** Variation rate of the FACT-G questionnaire scores, total and subscales, stratified by clusters.

			TOTAL SCORE		PHYSICAL WELL-BEING		SOCIAL/FAMILY WELL-BEING		EMOTIONAL WELL-BEING		FUNCTIONAL WELL-BEING	
		N	Mean	SD^a^	Mean	SD^a^	Mean	SD^a^	Mean	SD^a^	Mean	SD^a^
**Baseline**	cluster 1	160	53.04	9.64	17.19	4.63	17.61	4.10	6.58	2.28	11.66	5.24
	cluster 2	21	52.86	9.34	17.33	3.53	16.57	5.16	7.24	2.95	11.71	4.63
	cluster 3	66	60.33	10.07	17.18	4.05	19.94	4.13	10.47	3.54	12.74	4.68
	cluster 4	19	60.44	11.92	18.79	3.68	21.12	4.52	7.79	3.31	12.74	5.38
**1-month follow-up**	cluster 1	160	63.76	7.56	18.53	4.18	17.34	3.55	11.91	2.17	15.98	4.36
	cluster 2	21	63.62	7.26	18.43	3.47	17.81	4.81	11.14	2.13	16.24	3.66
	cluster 3	66	65.32	7.85	18.74	3.80	19.36	3.34	10.76	2.00	16.45	4.76
	cluster 4	19	68.72	7.77	20.47	3.72	20.51	4.61	11.74	2.68	16.00	4.61
**Variation rate**	cluster 1	160	0.22	0.12	0.08	0.09	-0.02	0.11	0.44	0.19	0.30	0.20
	cluster 2	21	0.23	0.16	0.06	0.04	0.09	0.15	0.34	0.27	0.30	0.18
	cluster 3	66	0.09	0.09	0.09	0.07	-0.03	0.10	0.01	0.34	0.23	0.16
	cluster 4	19	0.16	0.13	0.08	0.06	-0.04	0.09	0.29	0.41	0.23	0.19
*p*			< .001*		0.417*		< .001*		< .001*		0.056*	

*Kruskall Wallis test: Variation rate among clusters.

^a^SD: Standard deviation.

Statistically significant differences were detected for the change rate score of the FACT-G according to cluster (KW = 51.84; *p*<0.001), with the lowest rate score in cluster 3. According to the overall scores, we detected a greater change rate score in cluster 1 (0.22±0.12) and cluster 2 (0.23±0.16), and lower rate scores in cluster 4 (0.16±0.13) and cluster 3 (0.09±0.09) (KW = 47; *p*<0.001). Further, we observed that the mean initial scores for clusters 1 and 2 were the lowest in the ensemble of data. S3 Table displays the progression of the FACT-G scores and statistical significance of over-represented characteristics in the clusters.

## Discussion

The present study indicates that patients with cancer with symptoms of anxiety who received a protocol of abbreviated progressive muscle relaxation training [34] improved their perceived quality of life, as measured by the FACT-G. Patients who performed the technique experienced an increase in overall quality of life as well as in emotional, functional, and physical wellbeing. Our findings are consistent with previous reports that concluded CAM techniques (including relaxation techniques) can improve the quality of life of patients with cancer [11,18–25].

Beard et al. [22] investigated the effects of reiki and muscle relaxation in 54 patients with prostate cancer. Patients received two reiki sessions plus one muscle relaxation session per week for 8 consecutive weeks. The latter was also recommended to be practiced daily at home. Although those authors did not find differences in overall FACT-G scores, statistically significant improvements were detected in emotional wellbeing. Interestingly, in our study, the FACT-G emotional wellbeing subscale showed the greatest improvement. Andersen et al. [18] analyzed the effects of a 6-week program of exercises and muscle relaxation in 213 patients undergoing chemotherapy treatment. In that study, the combination of high and low intensity physical exercises and relaxation exercises did not produce significant changes in FACT-G scores [18]. Isa et al. [23,24] studied the effect of progressive muscle relaxation on anxiety in a group of 155 patients with prostate cancer. They found statistically significant improvements in quality of life at 4 and 6 months, measured with the 36-item Short Form Health Survey.

Another study on mindfulness also demonstrated improvements in quality of life, particularly in emotional wellbeing [39]. Ülger et al. [20] concluded that yoga practiced for 8 weeks by patients with cancer significantly improved energy levels, pain, emotional levels, sleep, social adaptation, and social skills. Another study involving yoga, in which women surviving breast cancer performed yoga five times a week for 6 months, showed improved quality of life and decreased abdominal perimeter [25]. Finally, Bar Sela et al. [11] published a study on complementary techniques (including relaxation) involving 163 oncological patients undergoing active treatment, and found significant improvements in quality of life (measured with the EORTC QLQ-C30 questionnaire) and in symptoms such as nausea, pain, and insomnia.

The strengths of our study include the inclusion of a large sample from different hospitals (multicenter study) and thorough training of research staff in performing the technique. However, potential limitations should also be considered. First, the lack of a control group represents the main limitation of this study, and does not allow determination that the results obtained were exclusively due to our intervention. However, a control/placebo group including patients with cancer was difficult to include from an ethical perspective. Second, recruitment for this study took place in hospitals via informative flyers, posters, and direct information provided by health professionals caring for the patients. Therefore, we do not know the total number of prospective participants who were informed of the study or the number of those who did not have access to this information. Third, we only evaluated short-term effects of the intervention and cannot determine long-term effects. Nevertheless, as several cancer types included in the present study have high mortality, longer term information may be difficult to obtain in future studies.

## Conclusions

Correct learning and regular use of progressive muscle relaxation techniques in the abbreviated version as described by Bernstein and Borkovec [34] contributes to short-term improvements in the perceived quality of life of patients with cancer.

## Supporting information

S1 Trend checklist TREND statement checklist. (PDF)

S1 Fig Dendrogram distribution of clusters based on the Functional Assessment of Cancer Therapy-General questionnaire. (TIFF)

S2 Fig Graphical representation of the distribution of clusters. (TIFF)

S3 Fig Variation of the overall Functional Assessment of Cancer Therapy-General questionnaire. (TIFF)

S4 Fig Variation of Functional Assessment of Cancer Therapy-General questionnaire subscales. (TIFF)

S1 Table Evolution of items of Functional Assessment of Cancer Therapy-General questionnaire subscales after relaxation intervention. (DOCX)

S2 Table Analysis of all existing developments in the sample. (DOCX)

S3 Table Variation of Functional Assessment of Cancer Therapy-General questionnaire and the statistically significant over-represented characteristics of the clusters. (DOCX)

S1 Data Database contains all of the data necessary to replicate all analysis in this study. (XLSX)

S1 Protocol Trial study protocol in English. (DOCX)

## Abbreviations

CAM: Complementary and alternative medicine
FACT-G: The Functional Assessment of Cancer Therapy-General
