# Supplementary figures and images for: The impact of muscle relaxation techniques on the quality of life of cancer patients, as measured by the FACT-G questionnaire
Source: PLoS One. 2017 Oct 19;12(10):e0184147. doi: 10.1371/journal.pone.0184147 (PMC5648131; doi:10.1371/journal.pone.0184147)

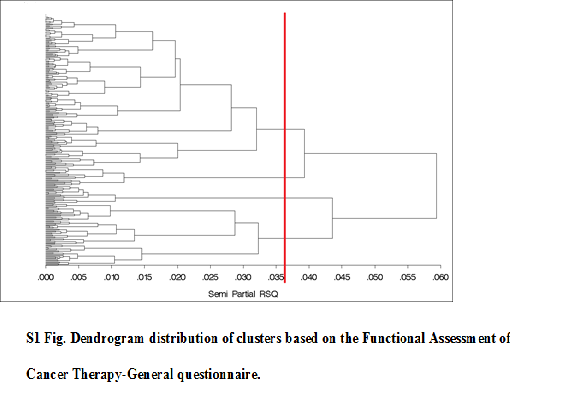

Supplement: S1 Fig — (TIFF) [file pone.0184147.s002.tiff]

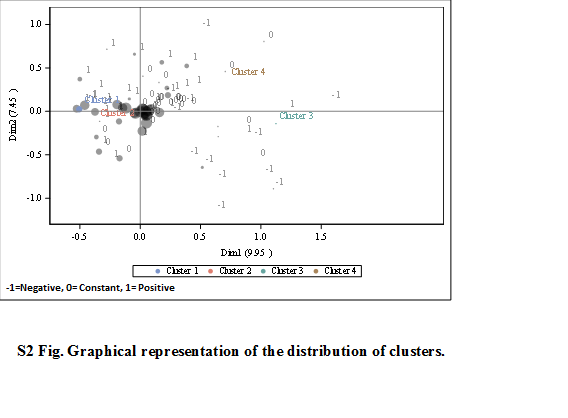

Supplement: S2 Fig — (TIFF) [file pone.0184147.s003.tiff]

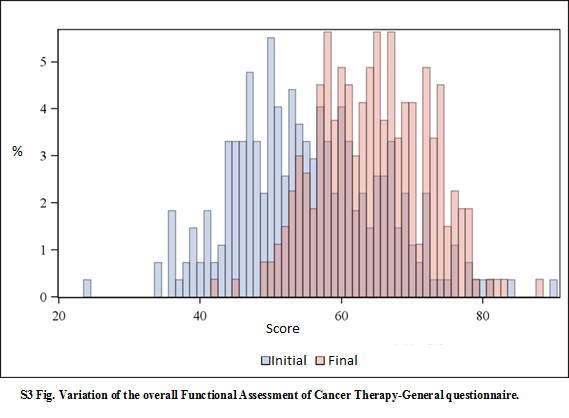

Supplement: S3 Fig — (TIFF) [file pone.0184147.s004.tiff]

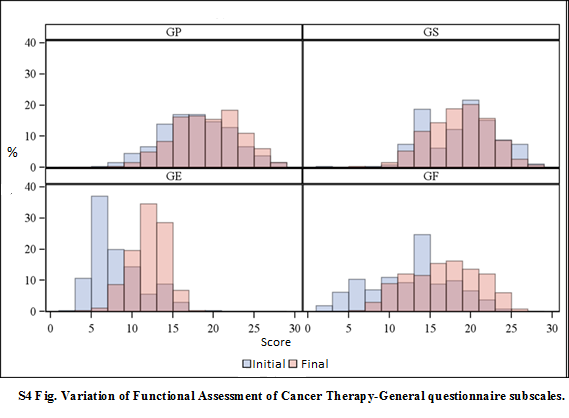

Supplement: S4 Fig — (TIFF) [file pone.0184147.s005.tiff]
