# Supplementary material for: The impact of muscle relaxation techniques on the quality of life of cancer patients, as measured by the FACT-G questionnaire
Source: PLoS One. 2017 Oct 19;12(10):e0184147. doi: 10.1371/journal.pone.0184147 (PMC5648131; doi:10.1371/journal.pone.0184147)
Supplement: S1 Table — (DOCX) [file pone.0184147.s006.docx]

|  | **EVOLUTION** | | | | | | | | | | | | |  |
| --- | --- | --- | --- | --- | --- | --- | --- | --- | --- | --- | --- | --- | --- | --- |
|  |  | **Negative** | | | | **Constant** | | | | **Positive** | | | | |
|  |  | **N** | **%** | **Mean difference** | **Overall average difference** | **N** | **%** | **Mean difference** | **Overall average difference** | **N** | **%** | **Mean difference** | **Overall average difference** |  |
|  |  |  |  |  |  |  |  |  |  |  |  |  |  |  |
|  |  |  |  |  |  |  |  |  |  |  |  |  |  |  |
| **PHYSICAL WELL-BEING** | GP1 | -- | -- | -- | -- | 141 | 53.01 | 0.91 | 8.48 | 125 | 46.99 | 1.94 | 9.85 |  |
|  | GP2 | 17 | 6.39 | 0.76 | 7.82 | 236 | 88.72 | 1.40 | 9.14 | 13 | 4.89 | 2.23 | 10.56 |  |
|  | GP3 | -- | -- | -- | -- | 244 | 91.73 | 1.31 | 9.03 | 22 | 8.27 | 2.41 | 10.21 |  |
|  | GP4 | -- | -- | -- | -- | 244 | 91.73 | 1.22 | 8.98 | 22 | 8.27 | 3.41 | 10.75 |  |
|  | GP5 | -- | -- | -- | -- | 246 | 92.48 | 1.29 | 8.89 | 20 | 7.52 | 2.75 | 12.04 |  |
|  | GP6 | -- | -- | -- | -- | 198 | 74.44 | 0.95 | 8.38 | 68 | 25.56 | 2.69 | 11.30 |  |
|  | GP7 | 3 | 1.13 | 1.00 | 8.00 | 225 | 84.59 | 1.25 | 8.74 | 38 | 14.29 | 2.32 | 11.50 |  |
| **SOCIAL/FAMILY WELL-BEING** | GS1 | 32 | 12.03 | -0.62 | 11.22 | 209 | 78.57 | -0.44 | 8.45 | 25 | 9.40 | 1.77 | 12.05 |  |
|  | GS2 | 7 | 2.63 | -2.14 | 3.29 | 230 | 86.47 | -0.39 | 9.01 | 29 | 10.90 | 1.36 | 11.46 |  |
|  | GS3 | -- | -- | -- | -- | 231 | 86.84 | -0.48 | 8.78 | 35 | 13.16 | 1.29 | 11.40 |  |
|  | GS4 | 7 | 2.63 | -3.48 | 3.38 | 223 | 83.83 | -0.43 | 8.81 | 36 | 13.53 | 1.48 | 12.18 |  |
|  | GS5 | -- | -- | -- | -- | 224 | 84.21 | -0.50 | 8.61 | 42 | 15.79 | 1.10 | 11.89 |  |
|  | GS6 | 175 | 65.79 | -1.06 | 8.40 | 61 | 22.93 | 0.78 | 10.30 | 30 | 11.28 | 2.42 | 10.95 |  |
|  | GS7 | -- | -- | -- | -- | 215 | 88.48 | -0.41 | 9.03 | 28 | 11.52 | 1.14 | 10.64 |  |
| **EMOTIONAL WELL-BEING** | GE1 | -- | -- | -- | -- | 103 | 38.72 | 3.67 | 8.79 | 163 | 61.28 | 3.99 | 9.34 |  |
|  | GE2 | -- | -- | -- | -- | 188 | 70.68 | 3.17 | 8.31 | 78 | 29.32 | 5.54 | 11.10 |  |
|  | GE3 | 95 | 35.71 | 0.86 | 5.67 | 56 | 21.05 | 4.09 | 9.74 | 115 | 43.23 | 6.23 | 11.68 |  |
|  | GE4 | 37 | 13.91 | -1.62 | 3.73 | 33 | 12.41 | 0.61 | 4.96 | 196 | 73.68 | 5.45 | 10.85 |  |
|  | GE5 | 45 | 16.92 | -0.58 | 3.38 | 43 | 16.17 | 1.86 | 7.17 | 178 | 66.92 | 5.47 | 11.05 |  |
|  | GE6 | 17 | 6.39 | 3.88 | 5.93 | 226 | 84.96 | 4.25 | 9.56 | 23 | 8.65 | 0.04 | 7.25 |  |
| **FUNCTIONAL WELL-BEING** | GF1 | -- | -- | -- | -- | 209 | 78.57 | 3.83 | 8.83 | 57 | 21.43 | 5.16 | 10.23 |  |
|  | GF2 | 9 | 3.38 | 2.33 | 6.28 | 200 | 75.19 | 3.90 | 9.82 | 57 | 21.43 | 5.14 | 7.15 |  |
|  | GF3 | 9 | 3.38 | 2.89 | 2.87 | 153 | 57.52 | 3.52 | 8.90 | 104 | 39.10 | 5.10 | 10.00 |  |
|  | GF4 | 31 | 11.65 | 1.61 | 3.97 | 52 | 19.55 | 3.48 | 7.02 | 183 | 68.80 | 4.72 | 10.60 |  |
|  | GF5 | 1 | 0.38 | 3.00 | 7.83 | 49 | 18.42 | 2.61 | 5.03 | 216 | 81.20 | 4.46 | 10.06 |  |
|  | GF6 | -- | -- | -- | -- | 213 | 80.08 | 3.88 | 8.87 | 53 | 19.92 | 5.04 | 10.15 |  |
|  | GF7 | -- | -- | -- | -- | 136 | 51.13 | 3.17 | 8.08 | 130 | 48.87 | 5.10 | 10.22 |  |
| **Mean difference**: Mean of the Score's difference obtained for all the items of each subscale (emotional status, personal functioning, general physical health state and social and family context).  **Overall average difference:** Mean of the Score's difference (for all the questionnaire's items). | | | | | | | | | | | | | | |

|  |
| --- |

**S1 Table. Evolution of items of FACT-G questionnaire subscales after relaxation intervention.**
