# Supplementary material for: The impact of muscle relaxation techniques on the quality of life of cancer patients, as measured by the FACT-G questionnaire
Source: PLoS One. 2017 Oct 19;12(10):e0184147. doi: 10.1371/journal.pone.0184147 (PMC5648131; doi:10.1371/journal.pone.0184147)
Supplement: S2 Table — (DOCX) [file pone.0184147.s007.docx]

| **Inertia and Chi-Square Decomposition** | | | | |
| --- | --- | --- | --- | --- |
| **Singular Value** | **Principal Inertia** | **Chi-Square** | **Percent** | **Cumulative Percent** |
| 0.37329 | 0.13934 | 555.98 | 9.95 | 9.95 |
| 0.32286 | 0.10424 | 415.92 | 7.45 | 17.40 |
| 0.31271 | 0.09779 | 390.18 | 6.98 | 24.38 |
| 0.29988 | 0.08993 | 358.82 | 6.42 | 30.81 |
| 0.29415 | 0.08652 | 345.23 | 6.18 | 36.99 |
| 0.28060 | 0.07874 | 314.16 | 5.62 | 42.61 |
| 0.27902 | 0.07785 | 310.64 | 5.56 | 48.17 |
| 0.27252 | 0.07427 | 296.32 | 5.30 | 53.48 |
| 0.26657 | 0.07106 | 283.52 | 5.08 | 58.55 |
| 0.25437 | 0.06471 | 258.18 | 4.62 | 63.17 |
| 0.24795 | 0.06148 | 245.31 | 4.39 | 67.57 |
| 0.24485 | 0.05995 | 239.20 | 4.28 | 71.85 |
| 0.23549 | 0.05546 | 221.27 | 3.96 | 75.81 |
| 0.23368 | 0.05461 | 217.89 | 3.90 | 79.71 |
| 0.22496 | 0.05061 | 201.92 | 3.61 | 83.32 |
| 0.21934 | 0.04811 | 191.96 | 3.44 | 86.76 |
| 0.21569 | 0.04652 | 185.63 | 3.32 | 90.08 |
| 0.20646 | 0.04263 | 170.08 | 3.04 | 93.13 |
| 0.19976 | 0.03990 | 159.22 | 2.85 | 95.98 |
| 0.17803 | 0.03170 | 126.47 | 2.26 | 98.24 |
| 0.15682 | 0.02459 | 98.12 | 1.76 | 100.00 |
| Total | 140.000 | 5586.00 | 100.00 |  |
| Degrees of Freedom = 9275 | | | | |
|  | | | | |

**S2 Table. Analysis of all existing developments in the sample**
