# Supplementary material for: The impact of muscle relaxation techniques on the quality of life of cancer patients, as measured by the FACT-G questionnaire
Source: PLoS One. 2017 Oct 19;12(10):e0184147. doi: 10.1371/journal.pone.0184147 (PMC5648131; doi:10.1371/journal.pone.0184147)
Supplement: S3 Table — (DOCX) [file pone.0184147.s008.docx]

**S3 Table. Variation of FACT-G questionnaire and the statistically significant over-represented characteristics of the clusters.**

|  | **Item** | **Evolution** | **N** | **% variable** | **% cluster** | ***P**** | **N Total** |
| --- | --- | --- | --- | --- | --- | --- | --- |
|  |  | **(%total)** |  | **in the cluster** | **in the variable** |  |  |
| **cluster 1 (N=160)** | GP1 | constant (53.01) | 101 | 63.13 | 71.63 | <.001 | 266 |
|  | GS1 | negative (12.03) | 25 | 15.63 | 78.13 | 0.019 | 266 |
|  | GS4 | positive (13.53) | 27 | 16.88 | 75.00 | 0.036 | 266 |
|  | GS7 | constant (80.83) | 155 | 96.88 | 72.09 | <.001 | 266 |
|  | GE2 | positive (29.32) | 56 | 35.00 | 71.79 | 0.009 | 266 |
|  | GE3 | positive (43.23) | 82 | 51.25 | 71.30 | <.001 | 266 |
|  | GE4 | positive (73.68) | 155 | 96.88 | 79.08 | <.001 | 266 |
|  | GE5 | positive (66.92) | 130 | 81.25 | 73.03 | <.001 | 266 |
|  | GE6 | constant (84.96) | 144 | 90.00 | 63.72 | 0.004 | 266 |
|  | GF2 | constant (75.19) | 132 | 82.50 | 66.00 | <.001 | 266 |
|  | GF4 | positive (68.80) | 131 | 81.88 | 71.58 | <.001 | 266 |
|  | GF5 | positive (81.20) | 137 | 85.63 | 63.43 | 0.018 | 266 |
| **cluster 2 (N=21)** | GS2 | constant (86.47) | 21 | 100.0 | 9.13 | 0.041 | 266 |
|  | GS6 | constant (22.93) | 10 | 47.62 | 16.39 | 0.008 | 266 |
|  | GS7 | positive (10.53) | 21 | 100.0 | 75.00 | <.001 | 266 |
|  | GE1 | constant (38.72) | 13 | 61.90 | 12.62 | 0.022 | 266 |
|  | GF4 | positive (68.80) | 19 | 90.48 | 10.38 | 0.017 | 266 |
| **cluster 3 (N=66)** | GP1 | positive (46.99) | 42 | 63.64 | 33.60 | 0.001 | 266 |
|  | GS1 | constant (78.57) | 59 | 89.39 | 28.23 | 0.008 | 266 |
|  | GS4 | constant (83.83) | 61 | 92.42 | 27.35 | 0.019 | 266 |
|  | GS7 | constant (80.83) | 60 | 90.91 | 27.91 | 0.010 | 266 |
|  | GE2 | constant (70.68) | 55 | 83.33 | 29.26 | 0.006 | 266 |
|  | GE3 | negative (35.71) | 34 | 51.52 | 35.79 | 0.002 | 266 |
|  | GE4 | constant (12.41) | 26 | 39.39 | 78.79 | <.001 | 266 |
|  |  | negative (13.91) | 31 | 46.97 | 83.78 | <.001 | 266 |
|  | GE5 | constant (16.17) | 16 | 24.24 | 37.21 | 0.034 | 266 |
|  |  | negative (16.92) | 25 | 37.88 | 55.56 | <.001 | 266 |
|  | GE6 | positive (8.65) | 13 | 19.70 | 56.52 | <.001 | 266 |
|  | GF2 | positive (21.43) | 29 | 43.94 | 50.88 | <.001 | 266 |
|  | GF3 | negative (3.38) | 5 | 7.58 | 55.56 | 0.045 | 266 |
|  | GF4 | constant (19.55) | 21 | 31.82 | 40.38 | 0.004 | 266 |
|  |  | negative (11.65) | 22 | 33.33 | 70.97 | <.001 | 266 |
|  | GF5 | constant (18.42) | 18 | 27.27 | 36.73 | 0.028 | 266 |
|  | GF6 | constant (80.08) | 60 | 90.91 | 28.17 | 0.007 | 266 |
|  | GF7 | positive (48.87) | 39 | 59.09 | 30.00 | 0.038 | 266 |
| **cluster 4 (N=19)** | GE1 | positive (61.28) | 19 | 100.0 | 11.66 | <.001 | 266 |
|  | GF1 | constant (78.57) | 19 | 100.0 | 9.09 | 0.009 | 266 |
| *Wilcoxon Signed Rank Test | | | | | | |  |
| % Variable in the cluster: % response with this feature in the cluster. | | | | | | |  |
| % Cluster in the Variable: % response with this feature in the cluster, relative to the total response with this feature. | | | | | | |  |
