# Supplementary material for: The impact of muscle relaxation techniques on the quality of life of cancer patients, as measured by the FACT-G questionnaire
Source: PLoS One. 2017 Oct 19;12(10):e0184147. doi: 10.1371/journal.pone.0184147 (PMC5648131; doi:10.1371/journal.pone.0184147)
Supplement: S1 Protocol — (DOCX) [file pone.0184147.s010.docx]

**PROGRESSIVE MUSCULAR RELAXATION IN THE CARE OF THE ANXIETY And THE PAIN IN THE ONCOLOGIC PATIENT**

PROTOCOL STUDY

1. INTRODUCTION

- 1. Introduction to the oncology.

1. Concept of *C áncer*.

The cancer is a group of illnesses in which the development of a tumor characterises by the uncontrolled proliferation of the cells cancerígenas that have lost, or present a deficit of control in the multiplicación or in the signals of apoptosis. Andsto comports that it produce the mitosis indefinitely causing the invasion of surrounding fabrics and/or the invasión to distance or metastasis(1,2).

The malignancy of a tumor comes determined by his capacity of invasion, so much to local level as to distance, however do not know with accuracy the mechanisms through which produces .

In the last years has accepted that the cancer is a genetic illness since his pathophysiology roots in the mutations of the material genéticor(1,3).

These alterations have to produce in those genes involved in the synthesis of proteins, that correspond to 10% of all the human genome. To his time, only can produce a development tumoral those mutations whose genes are involved in the proliferation, differentiation, apoptosis, angiogénesis and metastasis(1).

They are many the factors of risk involved so that it produce a tumor but in the actuality have not achieved identify all the producing mechanisms of cancer.

Doll And Peto^(4)^ consideraban in 1981 that the 80-90% dand the tumors produced like consequence of environmental factors, and therefore prevenibles, being the most important the tobacco, the diet, the occupation and the infections.

During long it considered this report, commissioned by the Office of the Congress of United States of Evaluation of Technologies, like reference to establish estimates of cancer and preventive sanitary politics in the American population. However , in the actuality the implication of the environmental factors considers mucho lower since in some cases the factors of risk superimpose (5,6).

Even, Tomasandtyou and Volguestein in the year 2015, published that only a third is atribuible to environmental or hereditary causes and that the greater part produces like consequence of random mutations in the replication of normal cells and therefore, and quote textualmente, to the “*bad luck”*(7) .

- - 1. Data epidemiológicos.

The cancer is one of the illnesses (or groups of illnesses) of greater importance in terms of public health in the western countries, in fact situates between the 5 first causes of death(8,9).

To international level the data epidemiológicos are registered by the International Agency for Research on Cancer (IARC) dependent agency of the World Health Organisation (WHO).

In the year 2015 published the relative data to the 2012 corresponding to 184 countries. In total, there was 14,1 million new cases and 8,2 million deaths.

The cancers more commonly diagnosed were of pulmonary origin (1,82 millions), mammary (1,67 millions) and colorrectal (1,36 millions). Regarding the mortality, the cancer of lung was the one who more deaths produced (1,6 million deaths), followedd the cancer of liver (745.000 muertes) and dthe cancer of stomach (723.000 deaths)(10).

In Spain, theS ociedad Spaniard of Oncology Médica (SEOM), edits every year these data using the most reliable information possible on incidence, prevalencia and mortality(11).

In both sexes the cancer more incident is the colorrectal (15%), the most mortal the one of lung (20,6%) and the more prevalente to the 5 years is the cancer of breast (17,9%).

In men the cancer more incident is the one of cancer of prostate (21,7%), the most mortal the cancer of lung (27,4%) and the more prevalente to 5 years the cancer of prostate (31,4%).

In women the greater incidence, mortality and prevalencia to 5 years is for the cancer of breast (29%, 15,5% and 40,8%, respectively).

The five more frequent tumors for both sexes are colorrectal, prostate, lung, breast and bladder.

These data vary inside the Spanish territory as it shows the map of the cancer in Spain regarding the last 20 years where indicates that the geographic patterns influence in the development of cancer of stomach, colorrectal, lung and bladder (12).

This is especially notable to establish sanitary politics adjusted and individualizadas in function of the risk of the different Spanish regions.

- 1. Introduction to the mental health
     1. Concept of *Mental Health.*

They are many the definitions that exist on the madness. However they are not so many those that exist on Mental Health. It is possible that given the times that run few people aventuren to consider *“mentally healthy”* or *“fully balanced”*. Perhaps it is because we consider the health like something dynamic and fluctuante no like something static and perfectly clear-cut.

The World Health Organisation (WHO) defined in 1946 the health in general like *“a state of complete physical welfare, psychic and social and no only the absence of illness”*(13).

However this concept is very wide and very difficult to reach, since attain the complete welfare in the three spheres is more typical of punctual circumstances that of a durable condition in the time.

Menninger Defines the Mental Health like “the adaptation of the human beings to the world and to the others with the maximum of efficiency and of happiness”(14).

This definition is not exenta of interpretations since again it goes back to insist in the ethereal of the health and besides includes the so longed for happiness.

If it is difficult to define the Mental Health the happiness is not less. Aristotle tried it considering it the *“very supreme”*, the end to the cual are allocated all our actions, the aim of the life of the human beings. But it concluded with that the happiness is individual for each human being and that the means to achieve it could not generalise.

Thus the definition of Menninger follows without leaving clear that it is the Mental Health.

The World-wide Federation of Mental Health reformuló the definition of the WHO: “the Mental Health would be the best possible state insided and the existent conditions, **a formal promise, reflexive and responsible to the humanigive considered like an all, basada in the free consent and in the respect to the individual and cultural differences" (15),**  that is to say, the situation of welfare psicofísico that can have depending on the circumstances that surround to the individual in each moment .

With all the previous can conclude that the Mental Health is inherent part of the physical health and that it is practically impossible to feel healthy if we do not enjoy of psychological welfare but that also is intimately related with the circumstances and with our capacity to adapt us to them.

All know people that suffering a grave physical illness emanate happiness and vice versa, people physically healthy and with an apparently full life that live intranquilos, nervous, sleep badly, etc. that is to say do not possess Mental Health.

This situation can extrapolate to the oncology. Patients often healthy *“”*, even asintomáticos, until a day leave to be it and after a medical query are included in the group of the *“patients”* and even *“gravely ill”*.

Attending to the previously commented definitions a paciente with Mental Health adapts to the new circumstances and achieves the psychic welfare. But lamentablembody this no always is possible and the patients, often suffer an important psychological stress (*distress* emotional).

- - 1. Data epidemiológicos.

The mental disorders present an important magnitude in the world-wide population. They are not exclusive of a special group if no that they can give in any social group of any population or country. Nevertheless, they are factors of risk some appearances like the low socioeconomic level, the conditions of life, the culture, the social politics and the social protection, the marginalisation, the violence or the maltreatment doméstico, especially in the woman(16).

It results difficult to quantify to level epidemiológico the impact of the mental disorders since often they find infradiagnosticados. Nevertheless, the WHO estimates that they are in increase what produces consequences to social level, economic and of the human rights. Thus it attributes them a character “*prioritario”* in the sanitary politics (17).

The Plan of Action on the Mental Health 2013-2020 elaborated by the WHO(16) and the Strategic Plan of Investigation Biomédica in Network of Mental Health 2014-2016(18) they collect the data epidemiológicos more notable regarding the mental disorders. However, these data epidemiológicos are relatively obsolete since in some cases date of more than ten years.

The WHO estimates that 1 of each 4 people, that is to say, 25% of the world-wide population will suffer a mental disorder along his life. Being in the actuality some 450 million people those that suffer a mental illness(19).

SAnd attribute him 13% of the total of years of life adjusted by disability (DALYS) that lose like consequence of enfermedades and injuries in the world(20).

The disability that produce the mental illnesses was recognised by the Convention on the rights of the people with discapacida^d(21)^, of the United Nations approved on 13 December 2006 in the Headquarters of the United Nations in New York. Andn she, signals that the people withand nfermedades mental, present a disability like consequence of the difficulty of the interaction with the people, and the barriers, regarding the attitude and the surroundings, hamper his full and effective integration in the society.

In Spain, the last data epidemiológicos relative to the mental illnesses collect in the proyecto ESEMeD-Spain of 2006(22). It situated the figures of prevalencia of mental disorders in 8,5% affecting to 19,5% of the people sometime of his life, being the most usual disorder the depression.

The last national and international recommendations insist in the importance to carry out politics preventivas(16,18,23).

In the field oncologic the patients often suffer psychological stress that defines like an emotional state of suffering in which they predominate the symptoms of anxiety and depression that can be accompanied in occasions of physical symptoms like sleeplessness and cefalea(24,25).It estimates that roughly a third of the patients with cancer suffer some type of mental disorder during the course of the active treatment(26,27) reaching near of 20% of patients with anxiety and 13% with depression(27). In both cases the prevalencia is greater in women(27).This deep psychological impact can perdurar in the time, even once finalised the treatment. By this reason, in a lot of countries the sanitary politics look for to detect the symptoms and like this can provide the necessary psychological cares, being the improvement of the validity and utility of the instruments of screening object of investigation in the actuality(28).

- - 1. The Disorders of Anxiety and the Diagnostic Nurse of Anxiety.

The anxious symptomatology can evolve to Disorders of Anxiety properly said , that describe in the Manual Diagnostic and Statistician of the Mental Disorders (DSM-5®), like disorders that share characteristics of fear and excessive anxiety. The fear is an emotional answer in front of an imminent threat whereas the anxiety is an answer anticipatoria. Both answers can solapar. They consider *“disorders”* when the fear or the normal anxiety prolong in the time, further of the appropriate periods. These disorders characterise by the restlessness or impatience, fatigabilidad easy, difficulty to concentrate or have the mind in white, irritability, muscular tension and alterations of the dream. SAnd differentiate in his origin being able to be been due to a concrete circumstance or be inespecíficos or induced by substances. In any case the symptomatology repeats and can coexistir with attacks of panic and even compulsive obsessive symptoms. It fits to stand out that the difficulty of relaxation is defined in the DSM-5® like a symptom of the Disorders ofTo nsiedad(29).

The possibilities of treatment are wide, from the pharmacology to the psicoterapia where include the technicians of relaxation that every time are winning more interest in the field of the Psiquiatría and of the Mental Health.

It treats of a multidisciplinary boarding where the professionals of infirmary take part of active form in the process to warn and take care to the patients with anxiety and/or fear.

The administration of these cares requires a planning and a scientific method that has gone developing from finals of the years sixty where began to use the first diagnostics of infirmary(30).

Cater cares through this concept pretends to boost the critical activity of thought which requires a critical reasoning and the utilisation of labels diagnósticas defined in the Taxonomia North American Nursing Diagnosis Association (NANDA)(31).

However the critical reasoning requires knowledges, technical skills and interpersonales and attitudes(32). From here, that no pueda establish a process diagnóstico standard, since this would drive us to inaccurate clinical trials.

In this point is the art and the science of the infirmary. Nevertheless, historically it has associated the take care with the infirmary and the cure with the medicine, leaving us the art and giving the science to the medicine.

This theoretical separation does not give in the practice thanks to the Process Nurse that allows us the identification and resolution of problems. By means of this process can value from the perspective nurse the different needs of the patient and from a point of view holístico choose the label diagnóstica timely nurse.

We endow to our cares of scientific rigour without forgetting the component of *“art”* that has take care and that does so special to the profession nurse.

The Process Nurse is a dynamic and systematic method to offer cares centred in the attainment of some results expected and, besides, is humanístico since it bases in the person, the family or the community. It divides in 5 stages: assessment, diagnose, planning, execution and evaluation of the care(32).

After the phase of assessment of infirmary and using the critical reasoning determine the labels diagnósticas timely, what in the process designates Diagnóstico Nurse: *“ …a trial about the answers of the individuor, family or community to the problems of health/real vital processes or potential. The diagnostics nurses provide the base of the selection of interventions nurses to reach the results of which the nurse is responsible”*(30)*.*

Later and of form individualizada establish those aims (results) to achieve and the clear-cut interventions to attain it. These aims and interventions are defined in the Classifications of Results and of Interventions of Infirmary (NOC and NIC)(33,34).

From the point of view of the Process Andnfermero results especially notable for this work the label diagnóstica of “Anxiety”. This label defines the Anxiety eat: “*Feeling wanders and intranquilizadora of unrest or threat accompanied of an autonomous answer (the origin of which with frequency is inespecífico or stranger for the person); feeling of aprensión caused by the anticipation of a danger. It is a signal of alert that warns of an imminent danger and allows to the person take medidas to face the threat”*(31)*.*

It presents a series of characteristics definitorias conductuales, affective, physiological and cognitive. This diagnostic of infirmary can evolve into the gunos cases to Anxiety In front of theM uerte clear-cut: “Feeling wanders and intranquilizante of unrest or fear caused by the perception of a real threat or imagined for the own existence”(31).

Disorders of Anxiety in oncology.

The complex process oncologic comports that in occasions the patients sufferor n important *distress* emotional(35-37). This *distress* defines like an emotional state of suffering in which they predominate the symptoms of anxiety and depression that can be accompanied in occasions of physical symptoms like sleeplessness and cefalea(24,25). estima That roughly a third of the patients with cancer suffer some type of mental disorder during thetr anscurso of the active treatment(26,27) reaching near of 20% of patients with anxiety and 13% with depression(27). In both cases the prevalencia is greater in women(27).

This deep psychological impact can perdurar in the time, even once finalised the treatment. By this reason, in a lot of countries the sanitary politics look for to detect the symptoms and like this can provide the necessary psychological cares, being the improvement of the validity and utility of the instruments of screening object of investigation in the actuality(28).

This *distress* emotional of the patient oncologic associates usually with the pain(37,38). By this reason, some psychotropic drugs are considered and administered like coadyuvantes of the analgesic treatment to be able to obtain a better control of the pain(38-40).

- 1. Introduction to the pain.
     1. Concept of *D smell.*

The International Association for the Study of Pain (IASP) defines the pain eat: *“A sensory and emotional experience unpleasant associated with damage tisular real or potential or described in terms of said damage”*(41).

It treats of an inherent quality to the life, coexist with him from the birth and our form to perceive it this modulated by our vital experiences, treats therefore of a subjective perception. Ands able to produce a big deterioration in the patient oncologic and limits seriously his quality life.

- - 1. Concepto of *D smell Orncológico.*

It does not exist a universally accepted definition to describe the *“pain oncologic”* however a high percentage of patients with cancer presents pain and, often, is not controlled(42-44). It estimates , that between 20% and 50% of the patients suffers it(45), and even, 90% when the illness is advanced(46).

A review of 46 studies published between the 1987 and the 2007 informed that 43.4% of average of the patients with cancer did not have controlled his pain being able to rise until 80%(44). Later another review inform that 31.8% of average of the patients with cancer had the bad pain controlled being able to rise until almost 70%(43). It estimates that near of the half of the patients does not have properly treated his pain(43,47).

His origin is variable, can be due to the own illness, to derivative appearances of the treatment or to comorbilidades associated(48).

It treats therefore of a fundamental appearance in the treatment of the patient oncologic and like this established it the 58º World-wide Assembly of the Health the WHO concluding that so much the relief of the pain like the palliative cares are prioritarios in the world-wide program of the cancer, thus it urged to the sanitary systems to establish programs of surveillance of the control of the pain(49).

Nevertheless, sor treatment and control is complejo. In fact the experts have difficulties to arrive to a consensus regarding the treatment of election(48,50) and thus the European Oncology Nursing Society proposes the reevaluación of the patient to ensure the efficiency of the treatment(48,51) and by, supposed, a treatment individualizado for each patient(42).

- 1. Introduction tol technical ace of relaxation.
     1. Concept of *R elajación.*

The relaxation is a state, achieved of conscious way and controlled, that characterises by a descent of the level of physiological and psychological activation. It drives to an increase of the calm, inner serenity or welfare(52).

- - 1. Types of technicians.

The technicians of meditation and/or relaxation can be defined like the group of technicians through which the patient reaches the harmony or inner peace(53). It treats of strategies autorreguladas that they can reduce the state of alert and attenuate the symptoms of anxiety(54).

These technicians centre in the interactions between the brain, the mind, the body and the behaviour with the intention to use the mind to improve the physical operation and promote the health. They look for the approach holístico and are designated Complementary and Alternative Therapies framed ofntro of the Medicine Integrativa(55).

Incluyen the meditation, the Yoga, the Tai Chi, exercises respiratoriyou, images guided, the hypnosis, the technicians of relaxation and the therapies expresivas like the music, the art, the dance, the biofeddback and the movement like therapy(55-57).

His complexity is very variable and some of them like the Yoga or the Tai chi require a training, a continuity and some skills motoras. Others, like the meditation or the imagery guided requires psychological skills.

In oncology, in the research of paliar the symptoms derived of the illness and/or treatment earn big importance the psychosocial interventions and the alternative and complementary therapies that they are increasingly frequent to improve his emotional state, fatigue, his quality of life and his adherencia to the treatment in patients that suffer different types of tumors(57-61).

- - 1. Technician of Progressive Muscular Relaxation of Jacobson.

The Technician of Jacobson is a technician of progressive muscular relaxation developed by Edmud Jacobson in the decade of 1920 and used fundamentally by psychologists, fisioterapeutas and skilled nurses in Mental Health.

The aim of the therapy is to free the muscular and physical tension to achieve a state of welfare that favour the decrease of the anxiety, the improvement of the quality of the dream and even of the pain.

The physiological base of this technical roots in that the neuromuscular hypertension is the base of the negative emotional states(62). Achieving the relaxation of the musculature will achieve the relaxation of the mind reaching a state of welfare that helps to control the states of anxiety.

The system musculo skeletal is interconnected through systems of fascias. These fascias constituted fundamentally by fabric conectivo experience changes of tension causing hipertonías that compensate with zones hipotónicas. Achieve the balance in all the system miofascial is the aim of the muscular relaxation.

The contraction of the skeletal muscular fibres drives to the feeling of tension that is the result of a complex interaction of the central nervous system and peripheral. According to Jacobson the muscular relaxation is incompatible with having thoughts or feelings that generate fear, anxiety or stress(62).

It established that between the body and the mind formed *a “circuit of effort”*(63) where the cerebral centres and the muscles worked together forming a neuromuscular circuit.

Of form very general relate the states of tension with the stress as well as the muscular relaxation with the mental *“relaxation”* properly said.

This increase of tension comes generated by an increase of the activity of the autonomous nervous system that involves the activation of the nice fibres or parasimpáticas, in the case of the stress, the first.

Walter Rudolf Hess, Nobel prize of Medicine in 1949 by mapear the areas of the brain wrapped in the control of the internal organs, defined the diencephalon like the cerebral location where carries out the coordination of the autonomous nervous system(64).

This system, divided in nice and parasimpático, often works reciprocally so that the activation of one supposes the suppression or modulation of the another.

Whereas the nice system is the system of alert struggles or huída the parasimpático is the one who drives to the relaxation and to the repair.

This was showed by Cannon, that in 1929 observed, in an essay with animal as it increased the cardiac frequency, the arterial tension, the flow of blood to voluntary muscles, the hiperglucemia and the pupillary dilatation in situations of stress or of activation of the nice producing the contrary in activation of the system parasimpático(65).

During the phase of relaxation produces a biological answer that minimises the activity of the nice system diminishing the consumption ofor xígeno and the cardiac frequency(66). The reduction of the skeletal muscular tone drives to a loss in the nice tone of the hypothalamus and, in consequence, to a predominance of the system parasimpático (67).

In other words the muscular relaxation comports an increase of the activity parasimpática to level of the hypothalamus. This situation induces a state of global relaxation that has showed efficiency for the treatment of the stress, the anxiety, the sleeplessness and even the hypertension and the painis of head (68,69).

Lundgren In 2006 related the anxiety generated by the fear to the dentist with the Technicians of Muscular Relaxation obtaining a decrease in the anxiety(70).

Although in principle it treat of a technician, the muscular relaxation is considered like an under control voluntary skill that it can turn into a persistent habit and that besides can be faster and deeper with the practice(71).

Jacobson Insists in the need to concentrate in the feelings developing the *“perception learnt”* so that the patient recognise the muscular tension and of this way can delete it(72).

With this relaxation achieves a substantial decrease of the anxiety and of the functional deterioration associated to some types of anxiety attaining a global improvement moderated in the life of the patient(71).

The literature suggests that in patients with cancer, produces an improvement of the symptoms like result of the elimination of the physical tension, emotional stress and the sleeplessness(73,74).

The efficiency of the therapy of relaxation to reduce the level of anxiety could be been due to the relaxation of the stimulation of the nice activity causing decrease of the arterial pressure, the cardiac frequency, the muscular tension and rhythm of the breath , as well as feelings to be calm and keeping the control(72).

They exist few studies that show the efficiency of these technicians but this has to mayormente to the heterogeneity of them ismos and of the own technical(57).

Nevertheless, the first metananálisis on the Technicians of Relaxation published in 2001 and concluded that had a positive effect in the adjust emotional, the functional adaptation and in the secondary symptoms to the treatment(75), without embrago, does not exist any study that relate the Technician of Jacobson specifically with the patients oncologics. Only, it studied his effect in the patient with cancer of prostate, being the positive results, obtaining a reduction of the anxiety, the stress and improvement of the quality of life (76,77).

In spite of this, the results aim to beneficial effects and, besides, are not described adverse effects, with which, are technical safe to combine with the farmacological treatment if there was him(53).

Tolgunos studies aim that the technicians in which it mediates the relaxation and diminish the anxiety, have collateral effects as it cann be the reduction of the nauseas(58,78,79) and of the pain(72,80-85).This is due to that the nauseas and vomits anticipatorios to the treatment characterise by pictures of increasing anxiety(86).

In summary, the relaxation has like general aim help to the patient to surpass the tension produced by the anxiety, the disorders of the dream and even pretends to help to control other symptoms like the pain or the nauseas.

The Technician of Jacobson treats of a technician that requires long, which diminishes the adherencia to the therapy, thus, developed back methods abbreviated of which the one of Bernstein & Borkovec of 1973 is one of the most known(87).

Although the base is the Technician of Jacobson there are some differences like the intensity of the contraction and the use of the suggestion.

In the method abbreviated consider the effect of the contraction pendular in such a way that what elder was this, greater will be the relaxation(87). However Jacobson considers the contraction like a half to cultivate the sensitivity of the individual(62,63) never like a half to achieve the relaxation(88).

Regarding the use of the suggestion, whereas Jacobson considers it unacceptable avoiding expressions of the type *“His extremities are heavy/toneless/relaxed”*(62), Bernstein & Borkovec plead for the indirect suggestion by means of expressions of the type *“Observe like his muscles feel more and more relaxed”*(87).

2. JUSTIFICATION

The padecimiento of an illness oncológica supposes often an alteration of the Mental Health of the people.

These alterations can be treated from different perspectives where include the farmacological therapy, the psychological interventions or the technicians of relaxation, between others.

In the field of the oncology the professionals of infirmary take part actively in this process trying provide the tools to the patients splitting of a plan of cares developed by means of the Process Nurse.

In spite of the heterogeneity of the studies published exist evidences that aim to an improvement in the symptoms derived of the disorders of anxiety. With the advantage that it treats of technicians no invasivas and no farmacological and therefore they lack risks and can be easily combinable with other treatments.

The studies are scarce and include different technical of relaxation, in this case want to centre us only in the Technician of Progressive Muscular Relaxation of Jacobson in his version abbreviated of Bernstein & Borkovec and isolate his effects of other treatments.

The difficulty of the study roots in the big quantity of variables that take part. Nevertheless, since they do not exist sufficient studies that have investigated the effect of the technicians of relaxation in the patients oncologics indispensable return the preparation of experimental studies controlled to elucidate that technical are more beneficial and which are the clinical characteristics of the patients that more can benefit of them.

3. HYPOTHESIS

1. Hypothesis general

The learning and use of the Technician of Progressive Muscular Relaxation of Jacobson in his version abbreviated reduces the anxious symptomatology and the pain improving therefore the quality of life of the patients oncologics.

1. Hypothesis secundarias

Andl consumption of drugs ansiolíticos, hypnotic, antidepressant and analgesics sees reduced in the patients that learn and use the technician of relaxation.

The prevalencia of patients oncologics that suffer disorders of anxiety is elevated in the actuality.

The degree of relaxation, concentration, command of the technician and confidence in his utility increase of significant form to measure that the patient practises the technician.

The utilisation of said technical influences positive and significantly on the nauseas and the quality of life in patients oncologics.

4. AIMS

1. Objective general

Andl objective of this study is to implement and valuesr a protocol of relaxation by means of the technician of Progressive Muscular Relaxation abbreviated by Bernstein & Borkovec in the patient oncologic with anxious symptomatology and pain.

1. Objective secundarios

Describe the consumption of drugs ansiolíticos, hypnotic, antidepressant and analgesics in the group control and group intervention.

Identify the prevalencia of patients oncologics that suffer disorders of anxiety in the group control.

Determine the degree of relaxation, concentration, command of the technician and confidence in his utility.

Determine the effect of said technical on the nauseas and the quality of life in patients oncologics.

5. MATERIAL And METHODS

1. Type of study.

It proposes a Quantitative Study of Intervention Cuasiexperimental Multicentric in which they participate 10 hospitals of the National System of Health.

1. Subjects of study

The group control is a cohorte aleatorizada by means of simple random sampling of patients, greater of 18 years, attended in the services oncologics of the University Marquis of Valdecilla, Santander (Cantabria), in the two previous years to the start of the study, that is to say, between 1 July of the 2012 and on 1 July of the 2014. descartan Those incomplete clinical histories or that do not collect the data to study. Through the grupo control only can compare with the group intervención the consumption of psychotropic drugs, analgesics and levels of pain since we do not have of the data of quality of life in the clinical histories.

The group intervención are the pertinent patients of the services oncologics of the hospitals involved. The criteria of inclusion and exclusion of the subjects of the group intervention are the following:

1. Criteria ofi nclusión:

All the patients oncologics, greater of 18 years, that refer anxious symptomatology: muscular tension, difficulty to conciliate the dream, attacks of fear or anxiety, etc., that find or begin the treatment of the process oncologic. The control of the pain considers a collateral effect to the technician with which does not consider sufficient criterion to be incluido in the study suffer pain.

1. Criteria ofand xclusión:

Although they are not described adverse effects in the utilisation of this technical it is necessary to take into account that in no case it supposes a substitute of the medical treatment besides exclude the patients that suffer hallucinations, deliriums or other symptoms psicóticos place that the exercises can drive to feelings extracorpóreas.

The participation in the study of the different centres is gradual and initiates in November of the 2014 and finalises in October of the 2015.

1. Intervention

After the catchment and the withfeeling informed, invited to the patients to participate in one sion of relaxation, individual or in group in function of the situation of the patient. In said session, sand instructed to the subjects on the Technician of Relation of Jacobson in his version abbreviated. The regoring dand the data made through the clinical history and of the own patient.

The protocol of relaxation this described in the Annex .

After the intervention sand provided them to the patients a reminder of the session where explains ba in detail by means of dibujos and text the technician (Annex ).

TodYou the researchers that carried out the intervention were formed on how make it and in all the relative to the selection of the participants, information of the same and collected of data with the end to unify criteria and reduce to the minimum the bias interexplorador. All the researchers contaron with a guide to drive the session of relaxation of the form more homogénea possible (Annex ).

1. Variables
2. Sociodemográficas.

Age, sex, medical centre of reference, familiar situation (civil state, children, familiar support) and academic training.

1. Related with the process oncologic.

Main medical diagnostic, date of the first query in the units oncológicas, type of treatment (chemotherapy, radiotherapy, biological therapies and hormonales) and his secondary effects.

Presence of pain, characteristic and control. They collect the date of start of the pain, the intensity, the handle and his efectividad. It considers “rescue” to the analgesic drug that guideline like adyuvante to the usual treatment, when this is not able to control the pain in a moment determinate.

The classification dand the analgesics did tiendiendo to the Analgesic Stairs ofthe Or MS for the control of the pain oncologic published in 1986(39).

1. Related with alterations of the mental health.

Characteristics of the consumption of antidepressants, hypnotic and ansiolíticos. Other no farmacological treatments to control his psychiatric symptomatology. They collect the date of startd the treatment, the handle and his andfectividad.

It considers “rescue” to the drug that guideline like adyuvante to the usual treatment, when this is not able to control the symptomatology in a moment determinate.

Regarding the guideline, considers “fixed” when the patient has prescribed a drug daily to control the symptoms, in a dose and determinate interval. It considers “discontinuous” when the patient has prescribed a drug, in a dose and determinate interval, that only consumes when they present the symptoms.

The anxiety or anxious symptomatology in function of his characteristicdefinitorias(31) it determines through the consumption of psychotropic drugs: ansiolíticos, hypnotic and antidepressant.

1. Related con the effect of the intervention.

Degree of previous anxiety to the technician and of back relaxation to the same, intensity of the pain, intensity of the nauseas, quality of life and degree of concentration, command and confidence in the development of the technician.

They collect data on the different anxious symptoms that suffer taking into account the intensity, his handle and the effect of the technician on them.

You tie to make the intervention and when finalising the follow-up values the quality of life.

The fascicles of collected of data (CRD) sand can consult in the Annex .

1. Instruments ofm edida
2. Scale of Verbal Qualification (ECV).

Valoración subjective of symptom/effect with a rank of 0 to 10. The intensity of pain following the recommendations of the European Society of Medical Oncology measures through the Scale of Verbal Qualification(47), where “0” is the absence of pain and “10” the maximum bearable pain.

1. Subjective effectiveness of the farmacological treatment analgesic, antidepressant, ansiolítico and hypnotic perceived by the patient regarding the control of the symptoms by means of four items.

Control dand the symptoms, do not control the symptoms, controlan partially the symptoms, does not collect the data.

1. Questionnaire of Quality of Life (FACT-G)(89).

This system of measurement, is developing from the year 1987. It initiated with the creation of a generic basic questionnaire called the Functional Evaluation of the Therapy of the General Cancer (FACT-G). The FACT-G (now in version 4) is a compilation of 27 items of general questions divided in four commands of quality of life principales: the physical and social welfare, welfare of the family, the emotional welfare and the functional welfare. It considers appropriate for his use with the patients with any type of cancer, and also has used and validated in other chronic illnesses (for example, HIV / SIDA and the multiple sclerosis) and in the general population (using a version ligeramente modificada). (Annex ).

1. Source of data

The collected of the data made by means of a CRD designed ad hoc for the group control and the group intervention (Annex ).

It used the clinical history of the patient in format paper and/or electronic for the data of the group control and intervention. The collected of data during the sessions of relaxation realizor by means of the autocumplimentación of said leaf by part of the subject to study. Nevertheless, contaron with the presence of the researcher that directs the session to resolve any doubt that could arise.

In the post intervention recogieron the data telephonically by part of a researcher, always the same, with the end to minimise the bias interexplorador.

1. Size muestral

The bibliography is scarce and by this reason have calculated the size muestral receiving us to the supuesto of maximum indeterminación (*p*=0,05), with an interval of confidence of 95% and a maximum error of the 5% of 400 people.

1. Plan of work.

The hospitals participants in this study have a team of professionals of infirmary that coordinates the project in each centre. Nevertheless, it has the collaboration of other members of the multidisciplinary team that loans services to the patient oncologic.

Once made the protocol and obtained the premisos pertinent proceeded to the presentation of the project in the units and to the professionals involved. Later, formor to all those researchers that participaron in the intervention, in all the relative to the technician, selection of participants, information and collected of data. After the period of training, whose aim was to achieve homogenise criterios, made the estudio pilot. This study consisted in the evaluation of the protocol of investigation in at least 15 participants of the group control and 15 participants of the group intervention of all the hospitals, with the end to amend possible errors in the intervention and collected of data. To favour the catchment of patients distribute in the units involved an informative triptych (Annex ).

The patients that entraron to form part of the study (group intervención), after consenting of form informed by writing, carryon to cape a session of relaxation following the guidelines of the protocol designed to such effect. His date and schedule of realisation was flexible and to suit between the two parts, researchers and participants. The collected of data hizo mensualmente through the clinical history and in the session of relaxation. Later the patient was subjected to a weekly telephone follow-up during a month.

Regarding the group control, after the pilot study and once determined the sample recogieron the data from the clinical history.

The inclusion of the data in the databases was gradual to measure that went collecting.

When finalising the collected of data, made the timely statistical analysis and drafted a memory.

Finally these data were presented in the units involved and to the scientific community.

The graphic description of the chronology of the estugave is described in the Figure 2.

It appears 2. Cronograma Of work

1. Statistical analysis of the data.

For the statistical analysis has differentiated between categorical characteristics (those that serve to classify to the patients in groups or categories) and quantitative characteristics (those that can measure from a clear-cut scale).

It has made the univariant descriptive analysis for all the variables:

- In the case of the categorical variables have calculated the headcount and percentage of each category.
- In the case of the quantitative variables have calculated the basic descriptive statisticians (N, average, typical deviation, minimum, average, maximum and headcount of stray values).

They have made analysis bivariados to examine the relation of the variables collected according to the characteristic/group of interest (sintomatologíto anxious, dsmell, sex, group (control/intervención)):

- Variables categóricas: Headcounts and percentages, (and Interval of Confidence 95%, of the percentage of patients) of the variable for each group of interest. These descriptive results support with the proof Chi-squared of Pearson (or the test of reason of verosimilitudes (LR-Ratio test), or the Exact Test of Fisher as it was convenient) to contrast the association between the variable and the group.
- Quantitative variables: Sand present the basic descriptive statisticians (N, average, typical deviation, minimum, average, maximum) for each the group of interest. These results support with the no parametric proof of Kruskal-Wallis to contrast the equality of the variable between groups.

They estimated Odds Ratios raw and adjusted together with his Intervals of Confidence to 95%, to determine the association between the control of the pain and the consumption of psychotropic drugs.

With the aim to determine the evolution of the variables collected in the sessions along the 4 weeks, have adjusted models for each assessment according to the week and the initial assessment, incorporating the measures repeated of a same subject along the 4 weeks(90). They have calculated the estimates and IC of the average for each assessment and week, in addition to the contrasts adjusted by the correction of Tukey of the difference of assessment between 2 consecutive weeks.

Finally, it has analysed the evolution of the punctuation of Quality of Life of the patients of the group Intervention by means of the tax of evolution of the global punctuation and in each section:

Tax of evolution = (Final Punctuation – Initial Punctuation)/Initial Punctuation)

That it quantifies us the difference of the evolution with regard to the start. We observe that positive values correspond to people with a final punctuation higher that at the beginning of the sessions. They have calculated :

- The basic descriptive statisticians N, average, standard deviation, minimum, P25, average, P75 and maximum of the different taxes of evolution.
- Histogram and diagram of box to visualise all the punctuations of the Questionnaire of Quality of life.
- These results support with the no parametric test Signed Rank of Wilcoxon to contrast if the tax of evolution is distinct of 0, that is to say, if they exist differences in the punctuation of the Questionnaire of Quality of Life between the initial moment and final.

Finally, it has made an analysis of multiple correspondences(91) to detect groups of patients, of the group Intervention, with evolution of the Quality of similar Life.

To make this analysis have considered that in each item of the questionnaire there is a Negative evolution, Constante or Positive, according to the punctuation to the start and at the end. From these evolutions in the indexes have considered how variable active, those indexes in what each evolution was present in more than 5% of the patients.

From this analysis, have obtained 21 factors that summary all the existent evolutions in the sample. With the most explanatory factors have made biplots including variable active (that they take part in the analysis) and illustrative variables (no included in the analysis) with the purpose to visualise possible groups of patients with similar characteristics.

From the 21 factors obtained in the analysis of correspondences has made a classification to group the similar answers. They have defined 4 groups/clústeres of patients with similar answers.

Once obtained the 4 groups has analysed which assessments, of the items of the questionnaire of quality of life, have a distinct proportion of the proportions of the sample. For each clúster, item of the Questionnaire of Quality of Life and type of evolution has calculated :

- % Of people with this evolution in the clúster.
- Number of people with this evolution in the clúster, with regard to the total number of people with this evolution in the sample.
- It has calculated the probability that the percentages of answers with each characteristic in the sample and in the clúster are equal, by means of the distribution Hyper-geometrical.

Finally:

- It has made the analysis bivariado of the tax of evolution of the questionnaire of Quality of Life according to the clúster. This analysis has made so much as a whole, as by block.
- Outlined of the clúster, concerning all the characteristics of the patients.

The statistical analysis has been made with the software: SAS v9.3, SAS Institute Inc., Cary, NC, USES. The statistical decisions have made with a level of significance of a 0,05.

1. Ethical considerations and himgales

This project is approved by the Committees of Ethical and corresponding Clinical Investigation to each hospital attending to the valid legislation (Annex 8).

It has been classified by part of the Spanish Agency of Medicines and Sanitary Products (AEMPS) in base to the valid legislation like “Observational Study No Postautorización (No-Epa)” (Anexor ).

Besides, attending to the rule of some hospitable centres, obtained lors pertinent permissions (Annex).

The paciente was informed, consintior by writing the participation in the project and the information was treated of confidential form as it regulates in the Regulatory Basic Law of the Autonomy of the Patient and of Rights and Obligations in Matter of Information and Clinical Documentation (Law 41/2002 of 14 November). According to this Law, this consent podríto be revoked incualq uier moment without that this affected to the quality of the cares received by the patient (Annex ).

The documentation for the patient is drafted in Spanish, nevertheless also had of the information in Catalan (Annex).

The data were treated of anonymous and confidential form with arrangement to the Organic Law 15/1999, of 13 December, of Data protection of Personal Character.

Hardnte all the study respetaron the ethical principles for the medical investigations in human beings as it collects in the Statement of Helsinki adopted in the 18ª Assembly of the World-wide Medical Association(92), likewise, respetaron the basic principles of the bioética:

Autonomy: The participation in the study is free and voluntary. The no participation will not suppose in no case a damage in the cares of the patient. It provided an informative leaf to the patient on the characteristics of the study and went of forced fulfillment the obtaining of a consent informed by writing. This consent was signed once that check that the patient comprised all the relativor to his participation and consented freely participate. In any case this podría be revoked anytime by part of the patient.

No maleficencia: Through this investigation do not derive damages neither for the patient neither for third.

Beneficencia: By means of this investigation pretends improve the cares that are catering to the patients. Therefore, the final aim is not another that benefit to the patients with the best cares and to the society using the resources of responsible form and justified.

Justice: The identity of the patient will be salvaguardad at all times. The results and all the derivative information of the investigation, will remain custodiada by the researcher. The result of and sta investigation will allow to cater cares of greater quality and use the resources of the society of responsible form.

1. Available means

The study required the utilisation of a space to develop the technician of relaxation. The hospitals involved disponían of the infraestructura necessary. The study had the support of the University of Cantabria and the University Rey Juan Carlos of Madrid. Explaining therefore with the software for the treatment and analysis of the data as well as the necessary advice.

TOTAL: 23.830€

6. BIBLIOGRAPHIC REFERENCES

1. Cruz Hernandez JJ, Rodríguez Sanchez CA, Of the Ship Morillo And. Clinical oncology. 5ª ed. Madrid: Group Classroom Medica; 2012.

2. González Baron M. Treaty of Clinical Oncology. 3ª ed. Madrid: Medical Moment Iberoamericana; 2010.

3. Boticario Boticario C, Cascales Narrow M. Cellular growth and cancer. Madrid: Uned; 1995.

4. Doll R, Peto R. The Cause of cancer: quantitative estimates of avoidable risks of cancer in the United States today. J Natl Cancer Inst 1981;66(6):308.

5. Parkin DM, Boyd L, Walker LC. The fraction of cancer attributable to lifestyle and environmental factors in the UK in 2010. Br J Cancer 2011;105 Suppl 2(S2):2.

6. Blot WJ, Tarone RE. Doll and Peto's Quantitative Estimates of Cancer Risks: Holding Generally True for 35 Years. J Natl Cancer Inst. 2015;107(4):djv044.

7. Tomasetti C, Volgestein B. Variation in cancer risk among. Science 2 2015;347(6217):81.

8. Rosell Coast R, Abad Esteve To, Monzo Planella M, Molina Medina F. Oncology Medica. Madrid: Ergon S.A.; 1995.

9. WHO: The 10 main causes of demise in the world 2014 [Internet]. [Consulted the 11/06/2015] Available in: http://www.who.int/mediacentre/factsheets/fs310/es/.

10. Ferlay J, Soerjomataram I, Dikshit R, Eser S, Mathers C, Rebel M et al. Cancer incidence and mortality worldwide: Sources, methods and major patterns in GLOBOCAN 2012. Int J Cancer 2015;136(5):86.

11. Spanish society of Medical Oncology (SEOM). The Figures of the Cancer in Spain 2014. SEOM; 2015.

12. López-Abente G, Aragonese N, Pérez-Gómez B, Pollán M, García-Pérez J, Ramis R et al. Time trends in Municipal distribution patterns of cancer mortality in Spain. BMC Cancer 2014 Jul 24;14:535.

13. WHO. Preamble of the Constitution of the World-wide Organisation of the health, International Sanitary Conference, New York, June 19 – July 22. 1946

14. Menninger K. The Vital balance. New York: Viking Press; 1963.

15. World-wide federation of Mental Health (FMSM). Mental health and World-wide Citizenship. 1948.

16. WHO. Plan of Action on the Mental Health 2013-2020.

17. WHO. 65ª World-wide Assembly of the Health. World-wide load of mental disorders and need that the sector of the health and the social sector answer of integral way and coordinated to scale of country. Ginebra;2012.

18. Centre of Investigation Biomédica in Network of Mental Health. Strategic plan 2014-2016.

19. World Health Organization Regional Office for Europe. Mental health:facing the challenges,building solution. Copenague;2005.

20. Murray CJ, Lopez AD. Global health statistics. Cambridge, MA, Harvard School of Public Health, (Global Burden of Disease and Injury Series, vol. II), 1996.

21. General assembly of United Nations.International convention on the Rights of the People with Disability. New York; 2006.

22. Haro JM, Palacín C, Vilagut G, Martínez M, Bernal M, Luque I. [Prevalence of Mental disorders and associated factors: results from the ESEMeD-Spain study].[Article in Spanish]. Med Clin (Barc). 2006 Apr 1;126(12):445-51.

23. WHO. Prevention of the Mental Disorders. Effective interventions and options of politics. Ginebra; 2004.

24. They gild DM. Psychological distress Ace to nurse-sensitive outcome. In Gild DM Nursing Outcomes The State of the Art. 2nd ed. Sudbury USES: Jones & Bartlett Learning; 2011

25. Drapeau To, Marchand To, Beaulieu-Prévost D. Epidemiology of Psychological Distress. In: LAbate PL, editor. Mental Illnesses - Understanding, Prediction and Control. Rijeka: InTech; 2012. p. 155–34

26. Singer S, Give-Munshi J, Brähler And. Prevalence of Mental health conditions in cancer patients in acute care--to put-analysis. Ann Oncol. 2010 May;21(5):925-30. doi: 10.1093/annonc/mdp515.

27. Linden W, Vodermaier To, Mackenzie R, Greig D. Anxiety and depression after cancer Diagnosis: prevalence rates by cancer type, gender, and age. J Affect Disord. 2012 Dec 10;141(2-3):343-51. doi: 10.1016/j.jad.2012.03.025.

28. Salmon P, Clark L, McGrath And, Fisher P. Screening for psychological distress in cancer: renewing the research Diary. Psycho‐Oncology 2015 March;24(3):262-268.

29. American Psychiatric Association. DSM-5. Manual Diagnostic and Statistician of the Mental Disorders. Madrid:Panamericana; 2014.

30. Gordon M. I diagnose Nurse. Process and Application.3ª ed. Madrid: Mosby/Doyma Books; 1996.

31. NANDA International. Nursing Diagnoses 2015-17: Definitions and Classification: Wiley-Blackwell; 2014.

32. Alfaro R. Application of the process nurse. Boost the care in collaboration. 5ª ed. Barcelona: Elsevier; 2003.

33. Moorhead S, Johnson M, Mass ML, Swanson And et al. Classification of resulted infirmary (NOC) : measurement of results in health. Barcelona: Elsevier; 2014.

34. Bulechek GM, Butcher HK, Dochterman JN, Wagner CM et al. Classification of interventions of infirmary (NIC). Barcelona: Elsevier; 2014.

35. Stanton To THE, Luecken LJ, MacKinnon DP, Thompson EH. Mechanisms in psychosocial interventions for adults living with cancer:Opportunity for integration of theory, research, and practice. J Consult Clin Psychol. 2013 Apr;81(2):318-35. doi: 10.1037/to0028833.

36. Mehnert To, Koch Or, Schulz H, Wegscheider K, Weis J, Faller H, et al. Prevalence of Mental disorders, psychosocial distress and need for psychosocial support in cancer patients - study protocol of an epidemiological multi-center study. BMC psychiatry 2012;12(1):70.

37. Cancer Pain (PDQ®). : PDQ Supportive and Palliative Care Publishing Board.; 2016.

38. Petkova M, Nikolov V, Galabova M, Petrova B. Psychological assessment of cancer patients with chronic pain. Procedia - Social and Behavioral Sciences 2010;5:421-425.

39. World Health Organization. Cancer pain relief and palliative care.Geneva: WHO 1986.

40. Mellar P. Davis. Opioids in Cancer Pain. 2nd ed. ed. GB: Oxford University Press; 2009.

41. The IASP Taxonomy. Classification of Chronic Pain.2011.

42. Portenoy RK. Treatment of cancer pain. Lancet. 2011 Jun 25;377(9784):2236-47.

43. Greco MT, Roberto To, Corli Or, Deandrea S, Bandieri And, Cavuto S et al. Quality of cancer pain management: an update of To systematic review of undertreatment of patients with cancer. J Clin Oncol 2014;32(36):54.

44. Deandrea S, Montanari M, Wets L, Apolone G. Prevalence of undertreatment in cancer pain. To review of published literature. Ann Oncol. 2008 Dec;19(12):1985-91. doi: 10.1093/annonc/mdn419.

45. Fischer DJ, Villines D, Kim I, et al.:. Anxiety, depression, and pain: differences by primary cancer. Support Care Cancer 18 (7): 801-10, 2010.

46. Goudas LC, Bloch R, Gialeli-Goudas M, Lau J, Carr DB. The epidemiology of cancer pain. Cancer Invest 2005;23(2):182-90.

47. Ripamonti CI, Santini D, Maranzano And, Berti M, Roila F; ESMO Guidelines Working Group. Management of cancer pain:ESMO Clinical Practice Guidelines. Ann oncol 2012;23(7):54.

48. Schug SA, Chandrasena C Pain management of the cancer patient. Expert Opin Pharmacother. 2015 Jan;16(1):5-15.

49. WHO.58ª World-wide Assembly of the Health. Prevention and control of the cancer. Ginebra;2005.

50. Davis MP, Walsh D, Lagman R, LeGrand SB. Controversies in pharmacotherapy of pain management. Lancet Oncol 2005;6(9):696-704.

51. Wengström And, Geerling J, Rustøin T.European Oncology Nursing Society breakthrough cancer pain guidelines. Eur J Oncol Nurs. 2014 Apr;18(2):127-31.

52. Megías-Lizancos F, Serrano Vine MD. Enfermeria In psiquiatría and mental health. Madrid: Editions DAE.; 2008.

53. Chen KW, Berger CC, Manheimer And, Forde D, Magidson J, Dachman L et al. Meditative Therapies for reducing anxiety: To systematic review and put-analysis of randomized controlled trials. Depress Anxiety 2012;29(7):62.

54. Krisanaprakornkit T, Sriraj W, Piyavhatkul N, Laopaiboon M. Meditation therapy for anxiety disorders. Cochrane Database Syst Rev 2009.

55. Or.S. National Center fort Complementary or Alternative Medicine https://nccih.nih.gov/health/integrative-health.

56. Dobos G, Altner N, Lange S, Musial F, Langhorst J, Michalsen To et al. [Mind-body medicine Ace to part of German integrative medicine].[Article in German]. Bundesgesundheitsblatt Gesundheitsforschung Gesundheitsschutz 2006;49:723-728.

57. Elkins G, Fisher W, Johnson To. Mind-body therapies in integrative oncology. Curr Treat Options Oncol 2010 December;11(3-4):128-40.

58. Bar-Sela G, Give us S, Visel B, Mashiach T, Mitnik I. The effect of complementary and alternative medicine on quality of life, depression, anxiety, and fatigue levels among cancer patients during activate oncology treatment: phase II study. The Indian Journal of Neurotrauma 2010 December;7(2):149-155.

59. Amichai T, Grossman M, Richard M. Lung cancer patients' beliefs about complementary and alternative medicine in the promotion of their wellness. Eur J Oncol Nurs 2012 December;16(5):520-527.

60. Bauml J, Langer CJ, Evans T, Garland SN, Desai K, Mao JJ. Does perceived Control predict Complementary and Alternative Medicine (CAM) use among patients with lung cancer? To cross-sectional survey. Support Care Cancer 2014 September;22(9):2465-2472.

61. Mahendran R, Lim HAS, So JY, Chua J, Lim , Ang IN, et al. Efficacy of To brief nurse-led pilot psychosocial intervention for newly diagnosed Asian cancer patients. Support Care Cancer 2015 August;23(8):2203-2206.

62. Jacobson And. Progressive relaxation. 2ª ed. Chicago: University of Chicago Press; 1938.

63. Jacobson And. Modern treatment of Tighten patients including the neurotic and depressed, with marry illustrations, follow-ups and EMG measurements. Ann Intern Med. 1970;72(5):770.

64. Hess WR, Brugger M. Functional organization of the diencephalon. New York: Grune & Stratton; 1957.

65. Cannon WB. Bodily changes in pain, hunger, fear and rage. New York.: Appleton; 1929.

66. Snyder M, Lindquist R. Complementary/alternative therapies in nursing. New York: Springer Publishing; 1998.

67. Gellhorn And, Kiely WF. Mystical states of consciousness: neurophysiological and clinical aspects. J Nerv Ment Dis 1972;154:399-405.

68. Grawe K, Donati R, Bernauer F. Psychotherapie im Wandel: Von der Konfession zur Profession. Göttingen: Hogrefe Publishing.; 2001.

69. King N. Progress in behavior modification. Abbreviated progressive relaxation. New York: Academic Press; 1980.

70. Lundgren J, Carlsson SG, Berggren Or. Relaxation versus cognitive therapies for Dental fear-to psychophysiological approach. Health Psychol, 2006;25(3):267-273.

71. Conrad To, Roth WT Muscle relaxation therapy for anxiety disorders: It works but how?.J Anxiety Disord 2007;21:264.

72. Payne RA. Relaxation Technique. To practical handbook for the health care professional. Barcelona: Paidotribo; 2002.

73. Kelly KM. Integrative therapies for children with hematological malignancies. Hematology Am Soc Hematol Educ Program 2009;307-312.

74. Ott MJ. Mind-body therapies for the pediatric oncology patient:matching the right therapy with the right patient. J Pediatr Oncol Nurs 2006;23(5):257.

75. Luebbert K, Dahme B, Hasenbring M. The effectiveness of relaxation training in reducing treatment-related symptoms and improving emotional adjustment in acute non-surgical cancer treatment: To it put-analytical review. Psychooncology 2001;10(6):502.

76. Isa MR, Moy FM, Abdul Razack AH, Zainuddin ZM, Zainal NZ. Impact of applied progressive deep muscle relaxation training on the level of depression, anxiety and stress among prostate cancer patients: to quasi-experimental study. Asian Pac J Cancer Prev 2013;14(4):2237-42.

77. Isa MR, Moy FM, Razack AH, Zainuddin ZM, Zainal NZ. Impact of applied progressive deep muscle relaxation training on the health related quality of life among prostate cancer patients To quasi experimental trial. Prev Med 2013;57(Suppl):37.

78. Burish TG, Lyles JN. Effectiveness of relaxation training in reducing adverse reactions to cancer chemotherapy. J Behav Med 1981;4(1):65-67.

79. Arakawa S. Relaxation to Reduces nausea, vomiting, and anxiety induced by chemotherapy in Japanese patients. Cancer Nurs 1997;20(0162-220; 5):342-349.

80. Nelson To, Hartl W, Jauch KW, Fricchione GL, Benson H, Warshaw To THE et al. The impact of music on hypermetabolism in criticalillness. Curr Opin Clin Nutr Metab Care 2008;11(6):790-794.

81. Coast To, Montalbano LM, Orlando To, Ingoglia C, Linea C, Giunta M et al. Music for colonoscopy: To single-blind randomized with-trolled trial. Dig Liver Dis 2010;42(12):871-876.

82. So X, Yowler CJ, Super DM, Fratianne RB. The efficacy of music therapy protocols for decreasing pain, anxiety, and muscle tension levels during burn dressing changes: to prospective randomized crossover trial. J Burn Care Animal 2010;31(4):590-597.

83. Pittman S, Kridli S Music intervention and preoperative anxiety:an integrative review. Int Nurs Rev 2011;58(2):163.

84. Buffum MD, Sasso C, Sands LP, Lanier And, Yellen M, Hayes To. Amusic intervention to Reduces anxiety before vascular angiogra-phy procedures. J Vasc Nurs. 2006 Sep;24(3):68-73.

85. Ulger Or, Yagli NV. Effects of Yoga on the quality of life in cancer patients. Complement Ther Clin Pract 2010 May;16(2):60-63.

86. Andrykowski MA, Redd WH.Longitudinal analysis of the development of anticipatory nausea. J Consult Clin Psychol. 1987 Feb;55(1):36-41.

87. Bernstein GIVES, Borkovec TD. Progressive relaxation training: To manual for the helping professions helping professions. Champaign, Illinois.: Research Press; 1973.

88. Lehrer PM, Batey DM, Woolfolk RL, Remde To, Garlick T. The effect of repeated Tighten-release sequences on EMG and self-report of muscle tension: an evaluation of Jacobsonian and post-Jacobsonian assumptions about progressive relaxation. Psychophysiology 1988;25:562-567.

89. Fact.org. [Internet]. [Consulted the 11/02/2014] Available in: http://www.facit.org/facitorg/questionnaires.

90. Verbeke G, Molenberghs G. Linear Mixed Models in Practice To SAS-Oriented Approach. Springer; 1997.

91. Greenacre M. The practice of the analysis of correspondences. 2nd ed. Barcelona: Rubes Publishing; (2008).

92. Statement of Helsinki of the World-wide Medical Association. Ethical principles for the medical investigations in human beings.18ª Medical Assembly World-wide.Helsinki, Finland;1964.

ANNEXES

**Annex 1. Certificate of the National Prize of Investigation in Infirmary.**

**Annex 2. Protocol of relaxation.**

PROTOCOL OF RELAXATION

Protocol of relaxation designed for the Technical *Project of Progressive Muscular Relaxation in the Prevention and Treatment of the Anxiety of the Patient Oncologic*. Based in the Technician of Progressive Muscular Relaxation of Jacboson (1938) in his metodo abbreviated by Bernstein & Borkovec (1973).

It establishes 1 session of learning that reunen the following caracteristicas.

Conditions of the room

Will carry out in a room enabled for such effect and will be directed by a nurse and/or fisioterapeuta.

Calm environment, the but silent possible.

Pleasant temperature.

Soft light.

I assent type chair or armchair the but comfortable possible.

Clothes of the patient

The but comoda possible. Avoid pieces very adjusted or any complement that it tighten like cinturon, buttons of the trousers, etc.

Group reduced

Of individual form or in groups from among 4 and 6 people to guarantee the deal but individualizado and a greater comfort of the patient when finding in a group reduced.

SESSION: 60 minutes of length

Theoretical class where will expose the characteristics but important of the technician, the aims of the same and the structure of the program of relaxation (10 min roughly).

Abdominal breath-diafragmatica: learning of the technician (5 minutes roughly).

Guideline of Relaxation

The position of start will be of seated the but comfortable possible in a chair, for this will facilitate him cushions to the patient. The back supported in the backrest, straight head, the hands on the thighs and the feet supported in the floor without crossing the legs.

We will begin the session with 5 breaths abdomino-diafragmaticas with the enclosed eyes.

To continuation will initiate the tension-relaxation of 16 muscular groups:

The phase of hard contraction between 5 and 7 seconds and the keyword will be “now” whereas the phase of hard relaxation between 30 and 40 seconds and the keyword will be “relax”.

Group 1: Form a fist with the dominant hand tightening the musculos of the hand and of the forearm.

Group 2: it Carry the elbow of the same arm against the arm of the chair activating the musculos of the arm.

Group 3 and 4 the same that the Groups 1 and 2 but for the upper extremity no dominant.

We divide the muscular groups of the face in three parts: Front, eyes and mandibula.

Group 5: Levente the eyebrows.

Group 6: it Tighten the eyes and frunza the nose.

Group 7: it Tighten the teeth and carry to backwards the commissure of the lips.

Group 8: Throw of the menton downwards and tighten the head against the support tightening the musculos of the neck.

Group 9: Carry the shoulders to backwards trying joint the escapulas for this is necessary that incorporate slightly to in front.

Group 10: Tighten the musculos abdominal entering the abdomen to inside.

Group 11: Tighten the dominant leg from the thigh contracting tensors and extensores of the knee simultaneamente.

Group 12: Make a flexión plant of the dominant ankle flexionando the foot to the floor.

Group 13: Make a flexión dorsal of the dominant foot flexionando the dominant ankle upwards.

Group 14, 15 and 16 repeat the Groups 11, 12 and 13 for the inferior extremity no dominant.

To finalise leaves between 1 and 2 minutes in silence disfrutrando of the feeling of complete relaxation.

Later it asks them to the patients that go moving slowly the hands and the arms, despues the legs, the head, the neck, that move in the chair and finally that it open the eyes.

Recommendations for his domicile.

Resolve doubts and difficulties.

Collected of data (15 minutes roughly) by means of leaf autocumplimentable by the patient.

**Annex 3. Reminder for patients.**

**REMINDER**


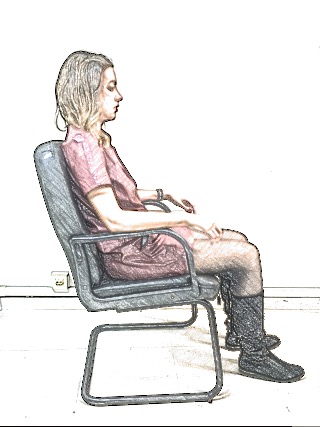
Plant in position in position of seated or tumbado, with the legs and the arms descruzados and with the head supported.

We recommend him that it use comfortable clothes or that desabroche the belt or buttons that opriman.

It is very important that concentrate in the exercises that go to make and that during the procedure do not think in situations that cause him stress or fear.

It leave that his body go relaxing to measure that advance.

BREATHE: To start with with the enclosed eyes go to make 5 abdominal breaths. Carrying all the air that inspire to our navel without moving the thorax.

MUSCULAR CONTRACTION: After the breaths, go to begin to contract the different muscular groups:

1. THE UPPER EXTREMITIES:

- It contract the FIST of his dominant hand during 7 seconds, afterwards, relax it during 15 or 30 seconds.
- To continuation, repeat it with his NO dominant FIST.

- It contract the FOREARM of the dominant side pushing against the reposabrazos of the armchair during 7 seconds, afterwards relax him during 15 or 30 seconds.
- It repeat the same exercise with the NO dominant FOREARM.

1. THE FACE: we Go to divide the muscular groups of the face in three parts: front, eyes and jaw.

- The first place raise the EYEBROWS during 7 seconds and afterwards leave that they relax during 15 or 30 seconds.
- Now it tighten the EYES and frunza the NOSE, again during 7 seconds and afterwards leave that it relax between 15 and 30 seconds.
- Finally, it tighten the TEETH and carry to backwards the commissure of the LIPS during 7 seconds and relax the face during 15 or 30 seconds.

1. THE NECK: Throw of the CHIN downwards and tighten the head against the headrest tightening the muscles of the neck during 7 seconds and afterwards relax the neck during 15 or 30 seconds.

1. THE SHOULDERS: it Carry the shoulders to backwards trying joint the escapulas/shoulders for this is necessary that incorporate slightly to in front during 7 seconds, afterwards relax them during 15 or 30 seconds.

1. THE ABDOMEN: it Tighten the abdominal muscles entering the abdomen to inside during 7 seconds, afterwards leave that it relax during 15 or 30 seconds.
2. THE INFERIOR EXTREMITIES:

- It tighten the dominant leg from the THIGH pushing against the chair during 7 seconds, afterwards leave it relaxed during 15 or 30 seconds.
- It repeat the same exercise with the another leg.

- It make a flexión of ANKLE as if it put on tiptoe pushing against the floor, again keep the contraction during 7 seconds and leave that it relax during 15 or 30 seconds.
- It repeat the same exercise with the another ankle.
- It make a flexión of ANKLE carrying the fingers to the knee during 7 seconds and relax him during 15 or 30 seconds.
- It repeat the same exercise with the another ankle.

END: Little by little it go moving slowly the hands and the arms, afterwards the legs, the head, the neck, move the trunk in the chair and finally that it open the eyes.

IMPORTANT NOTE

- It treats of isometric contractions in which there is not movement and do not have to be necessarily intense, thus **it does not have to to produce him pain** when pushing against the reposabrazos or when closing the fist, for example.
- The hard contraction some **7 seconds** whereas the relaxation can last between **15 and 30 seconds.** The time of relaxation can diminish it to measure that dominate the technician and when it do not have of the necessary time.

FAST RELAXATION And APPLIED

To measure that go practising will result him increasingly easy relax.

With the relaxation applied look for that *“it apply”* the technician learnt to situations of the daily life that produce him nervousness.

It will be able to adapt it to the circumstances in which it find and reduce the times of contraction-relaxation.

Of this way will be able to make it in any place although it do not have of the necessary time to make the complete technician, only will need to be seated and concentrate in the exercises.

If has some doubt do not doubt in contacting with the team researcher.

**Annex 4. Guide for the researcher on the session of relaxation.**

GUIA SESSION RELAXATION FOR RESEARCHERS

To continuation go to carry out a session of relaxation by means of the Technician of Jacobson in his version abbreviated.

Before beginning the session go to review the different exercises with the end to interrupt the less possible the development of the session: Fist, forearm, expensive, neck, shoulders, abdominal, leg and ankle. Once that know all the exercises explain in that it consists the abdominal breath and do a pair of attempts giving the timely indications.

The contractions will last roughly 7 seconds and do not have to be so intense that produce pain when tightening against the reposabrazos or when closing the fist for example.

The time of relaxation will oscillate between 15 and 30 seconds.

Plant in position in position of seated, with the legs and the arms descruzados and with the head supported. We recommend him that it use comfortable clothes or that desabroche the belt or buttons that opriman.

It is very important that concentrate in the exercises that go to make and that during the procedure do not think in situations that cause him stress or fear.

It leave that his body go relaxing to measure that advance.

To start with with the enclosed eyes go to make 5 abdominal breaths. Carrying all the air that inspire to our navel without moving the thorax.

We inspire-espiramos.

We inspire-espiramos.

We inspire-espiramos.

We inspire- espiramos.

Finally we inspire and espiramos.

Now we go to begin to contract the muscles.

1. In the first place we contract the fist of our dominant hand.

<1,2,3,4,5,6,7>

We relax it (explaining mentally until 15-30).

Now we contract the fist of our NO dominant hand.

<1,2,3,4,5,6,7>

We relax it (explaining mentally until 15-30).

1. It contract the forearm of the dominant side pushing against the reposabrazos of the armchair.

<1,2,3,4,5,6,7>

Now we go to leave that it relax (explaining mentally until 15-30).

We repeat the same exercise with the another arm. In the first place during 7 seconds go to contract the forearm of our no dominant hand.

<1,2,3,4,5,6,7>

We go to leave that it relax (explaining mentally until 15-30).

We go to divide the muscular groups of the face in three parts: Front, eyes and jaw.

1. The first place go to raise the eyebrows.

<1,2,3,4,5,6,7>

We leave the face relaxed (explaining mentally until 15-30).

1. We go to tighten the eyes and fruncir the nose.

<1,2,3,4,5,6,7>

We leave the face relaxed (explaining mentally until 15-30).

1. It tighten the teeth and carry to backwards the commissure of the lips

<1,2,3,4,5,6,7>

We leave the face relaxed (explaining mentally until 15- 30).

1. Throw of the chin downwards and tighten the head against the support tightening the muscles of the neck.

<1,2,3,4,5,6,7>

It leave that the neck relax (explaining mentally until 15-30).

1. It carry the shoulders to backwards trying joint the escapulas /shoulders for this is necessary that incorporate slightly to in front.

<1,2,3,4,5,6,7>

We leave that it relax (explaining mentally until 15-30).

1. It tighten the abdominal muscles entering the abdomen to inside.

<1,2,3,4,5,6,7>

We leave that it relax (explaining mentally until 15-30).

1. It tighten the dominant leg from the thigh pushing against the chair (contracting tensors and extensores of the knee simultaneously)

<1,2,3,4,5,6,7>

Now we leave that it relax (explaining mentally until 15-30).

It repeat the same exercise with the another leg.

<1,2,3,4,5,6,7>

We go to leave that it relax (explaining mentally until 15-30).

1. It make a flexión of ankle as if it put on tiptoe pushing against the floor (flexión plant of the dominant ankle flexionando the foot to the floor) .

<1,2,3,4,5,6,7>

We leave that it relax (explaining mentally until 15-30).

It repeat the exercise with the another ankle.

<1,2,3,4,5,6,7>

We leave that it relax (explaining mentally until 15-30).

1. It make a flexión of ankle as if it put on tiptoe pushing against the floor (flexión plant of the no dominant ankle flexionando the foot to the floor).

<1,2,3,4,5,6,7>

We leave that it relax (explaining mentally until 15-30).

It repeat the exercise with the another ankle.

<1,2,3,4,5,6,7>

We leave that it relax (explaining mentally until 15-30).

1. It make a flexión of ankle carrying the fingers to the knee (flexión dorsal of the no dominant foot flexionando the dominant ankle upwards).

<1,2,3,4,5,6,7>

We leave that it relax (explaining mentally until 15-30).

It repeat the exercise with the another ankle.

<1,2,3,4,5,6,7>

Finally, we leave that it relax (explaining mentally until 15-30).

Little by little they go moving slowly the hands and the arms, afterwards the legs, the head, the neck, the trunk in the chair and finally that it open the eyes.

**Annex 5. Fascicle of collected of data of the group control and of the group intervention.**

Leaf register group control NHC:

DATA SOCIODEMOGRÁFICOS

Date of birth: Sex: H/M Occupation:

Familiar situation: civil State: Married/Soltero/Divorced/Widower/Separated/Couple in fact Children IF NO Nºchildren: familiar Support: IF NO it does not collect

Medical data

It dates first query oncológica: Diagnostic oncologic main:

Treatment oncologic

Chemotherapy IF NO Type: (oral, endovenosa, intratecal, peritoneal): IQ IF NO

Radiotherapy IF NO

Therapy hormonal IF NO

Biological therapy IF NO

Notable secondary effects IF NO

-Type-relation-intensity (ej. Chemotherapy-Nauseas-Slight, Radiotherapy-Injury skin-Eritema)

|  |  |  |
| --- | --- | --- |
|  |  |  |
|  |  |  |
|  |  |  |
|  |  |  |
|  |  |  |
|  |  |  |
|  |  |  |

Treatment ansiolítico/hypnotic/antidepressant.

| ANSIOLÍTICOS | Treatment 1 | Treatment 2 | Treatment 3 | Treatment 4 |
| --- | --- | --- | --- | --- |
| Previous consumption to the diagnostic (IF it Does not date of start) |  |  |  |  |
| Type |  |  |  |  |
| Dose-Posología |  |  |  |  |
| Subjective effectiveness perceived by the patient ^1^ |  |  |  |  |

^1^.The patient refers control of the symptoms/the patient refers not controlling the symptoms/the patient refers to control partially the symptoms/ does not collect .

Other no farmacological treatments: (it dates start-type-frequency-subjective effectiveness perceived by the patient):

| HYPNOTIC | Treatment 1 | Treatment 2 | Treatment 3 | Treatment 4 |
| --- | --- | --- | --- | --- |
| Previous consumption to the diagnostic (If it Does not date of start) |  |  |  |  |
| Type |  |  |  |  |
| Dose-Posología |  |  |  |  |
| Subjective effectiveness perceived by the patient ^1^ |  |  |  |  |

^1^.The patient refers control of the symptoms/the patient refers not controlling the symptoms/the patient refers to control partially the symptoms/does not collect .

Other no farmacological treatments: (it dates start-type-frequency- subjective effectiveness perceived by the patient):

| ANTIDEPRESSANT | Treatment 1 | Treatment 2 | Treatment 3 | Treatment 4 |
| --- | --- | --- | --- | --- |
| Previous consumption to the diagnostic (IF it Does not date of start) |  |  |  |  |
| Type |  |  |  |  |
| Dose-Posología |  |  |  |  |
| Subjective effectiveness perceived by the patient ^1^ |  |  |  |  |

^1^.The patient refers control of the symptoms/the patient refers not controlling the symptoms/the patient refers to control partially the symptoms/ does not collect .

Other no farmacological treatments: (it dates start-type-frequency-subjective effectiveness perceived by the patient):

ANALGESIC TREATMENT

| ANALGESICS | Treatment 1 | Treatment 2 | Treatment 3 | Treatment 4 |
| --- | --- | --- | --- | --- |
| Pain (If it Does not date of start) |  |  |  |  |
| Location |  |  |  |  |
| Analog visual scale (EVA) |  |  |  |  |
| Treatment IF NO |  |  |  |  |
| Type |  |  |  |  |
| Dose-Posología |  |  |  |  |
| Rescue |  |  |  |  |
| Subjective effectiveness perceived by the patient^1^ |  |  |  |  |

^1^.The patient refers control of the pain/pain no controlled/pain partially controlled/does not collect .

Other no farmacological treatments: (it dates start-type-frequency-subjective effectiveness perceived by the patient):

PSYCHIATRIC DATA

Psychiatric PERSONAL ANTECEDENTS: IF it Does not date start: Diagnostic:

Treatment: Type-dose-posología:

After the diagnostic oncologic

Interconsulta To psiquiatría IF NO

Crisis of anxiety: income to skilled urgencies or primary attention: IF NO Those that:

Treatment psychologists, external psychiatrists, etc: IF NO Cual: Frequency:

Technicians of relaxation IF NO Cual: Frequency:

Complementary therapies IF NO Cual: Frequency:

Observations that the researcher consider notable:

Leaf register group intervention NHC:

DATA SOCIODEMOGRÁFICOS

Name: Surnames: Telephone: Hour of contact:

Date of birth: Sex: H/M Centre of origin: Occupation:

Familiar situation: civil State: Married/Soltero/Divorced/Widower/Separated/Couple in fact Children IF NO Nºchildren: familiar Support: IF NO it does not collect

Medical data

It dates first query oncológica: Diagnostic oncologic main:

Symptoms that motivate his inclusion in the study:

Treatment oncologic

Chemotherapy IF NO Type (oral, endovenosa, intratecal, peritoneal): IQ IF NO

Radiotherapy IF NO

Therapy hormonal IF NO

Biological therapy IF NO

Notable secondary effects IF NO

-Type-relation-intensity (ej. Chemotherapy-Nauseas-Slight, Radiotherapy-Injury skin-Eritema)

|  |  |  |
| --- | --- | --- |
|  |  |  |
|  |  |  |
|  |  |  |
|  |  |  |

Treatment ansiolítico/hypnotic/antidepressant.

| ANSIOLÍTICOS | Treatment 1 | Treatment 2 | Treatment 3 | Treatment 4 |
| --- | --- | --- | --- | --- |
| Previous consumption to the diagnostic (IF it Does not date of start) |  |  |  |  |
| Type |  |  |  |  |
| Dose-Posología |  |  |  |  |
| Subjective effectiveness perceived by the patient ^1^ |  |  |  |  |

^1^.The patient refers control of the symptoms/the patient refers not controlling the symptoms/ the patient refers to control partially the symptoms /does not collect .

Other no farmacological treatments: (it dates start-type-frequency-subjective effectiveness perceive by the patient):

| HYPNOTIC | Treatment 1 | Treatment 2 | Treatment 3 | Treatment 4 |
| --- | --- | --- | --- | --- |
| Previous consumption to the diagnostic (IF it Does not date of start) |  |  |  |  |
| Type |  |  |  |  |
| Dose-Posología |  |  |  |  |
| Subjective effectiveness perceived by the patient ^1^ |  |  |  |  |

^1^.The patient refers control of the symptoms/the patient refers not controlling the symptoms/ the patient refers to control partially the symptoms/ does not collect .

Other no farmacological treatments: (it dates start-type-frequency-subjective effectiveness perceive by the patient):

^1^.The patient refers control of the symptoms/the patient refers not controlling the symptoms/ the patient refers to control partially the symptoms /does not collect .

| ANTIDEPRESSANT | Treatment 1 | Treatment 2 | Treatment 3 | Treatment 4 |
| --- | --- | --- | --- | --- |
| Previous consumption to the diagnostic (IF it Does not date of start) |  |  |  |  |
| Type |  |  |  |  |
| Dose-Posología |  |  |  |  |
| Subjective effectiveness perceived by the patient ^1^ |  |  |  |  |

Other no farmacological treatments: (it dates start-type-frequency-subjective effectiveness perceive by the patient):

ANALGESIC TREATMENT

| ANALGESICS | Treatment 1 | Treatment 2 | Treatment 3 | Treatment 4 |
| --- | --- | --- | --- | --- |
| Pain (If it Does not date of start) |  |  |  |  |
| Location |  |  |  |  |
| Analog visual scale (EVA) |  |  |  |  |
| Treatment |  |  |  |  |
| Type |  |  |  |  |
| Dose-Posología |  |  |  |  |
| Rescue (Dose-Posología) |  |  |  |  |
| Subjective effectiveness perceived by the patient^1^ |  |  |  |  |

^1^.The patient refers control of the pain/pain no controlled/pain partially controlled/does not collect .

Other no farmacological treatments: (it dates start-type-frequency-subjective effectiveness perceive by the patient):

PSYCHIATRIC DATA

Psychiatric PERSONAL ANTECEDENTS: IF it Does not date start: Diagnostic:

Treatment: Type-dose-posología:

After the diagnostic oncologic

Interconsulta To psiquiatría IF NO

Crisis of anxiety: income to skilled urgencies or primary attention: IF NO Those that:

Treatment psychologists, external psychiatrists, etc: IF NO Cual: Frequency:

Technicians of relaxation IF NO Cual: Frequency:

Complementary therapies IF NO Cual: Frequency:

Observations that the researcher consider notable:

Relative data to the intervention

Date: Individual/Group: Number people

Quality of Life Scale Fact-G: initial DATA SESSION

Regarding the last 7 days:

|  | **GENERAL PHYSICAL STATE OF HEALTH** | **At all** | | **A bit** | **Something** | **A lot** | **Muchí- simo** |
| --- | --- | --- | --- | --- | --- | --- | --- |
|  |  |  | |  |  |  |  |
| GP1 | It is missing me energia................................................................ | 0 | | 1 | 2 | 3 | 4 |
| GP2 | Have nauseas.................................................................. | 0 | | 1 | 2 | 3 | 4 |
| GP3 | Because of my physical state, have difficulty to attend to the needs of my family……………..………….. | 0 | | 1 | 2 | 3 | 4 |
| GP4 | Have pain …………………………………………….. | 0 | | 1 | 2 | 3 | 4 |
| GP5 | They bother me the secondary effects of the treatment…... | 0 | | 1 | 2 | 3 | 4 |
| GP6 | I seat me ill(to)…………………………………… | 0 | | 1 | 2 | 3 | 4 |
| GP7 | I have to happen time put to bed(to)…………………… | 0 | | 1 | 2 | 3 | 4 |
|  | | | | | | | |
|  | **FAMILIAR And SOCIAL ENVIRONMENT** | **At all** | | **A bit** | **Something** | **A lot** | **Muchí- simo** |
|  |  |  | |  |  |  |  |
| GS1 | I seat me near(to) to my friendships.............................. | 0 | | 1 | 2 | 3 | 4 |
| GS2 | I receive emotional support by part of my family………. | 0 | | 1 | 2 | 3 | 4 |
| GS3 | I receive support by part of my friendships………………. | 0 | | 1 | 2 | 3 | 4 |
| GS4 | My family has accepted my illness………………… | 0 | | 1 | 2 | 3 | 4 |
| GS5 | I am satisfied(to) with the way in that it communicates my family about my illness…………………….  0 | 0 | | 1 | 2 | 3 | 4 |
| GS6 | I seat me near(to) to my couple (or to the person that is my main source of support)…………………………...  0 | 0 | | 1 | 2 | 3 | 4 |
| Q1  GS7 | *Without mattering his current level of sexual activity, answer to the following question. If it prefers to not to answer it, it mark this box*  *And it continue with the following section.* | | |  |  |  |  |
| GS7 | I am satisfied(to) with my sexual life………………… | | 0 | 1 | 2 | 3 | 4 |

|  | **EMOTIONAL STATE** | **At all** | | **A bit** | **Something** | **A lot** | **Muchí- simo** |
| --- | --- | --- | --- | --- | --- | --- | --- |
|  |  |  | |  |  |  |  |
| GE1 | I seat me sad................................................................. | | 0 | 1 | 2 | 3 | 4 |
| GE2 | I am satisfied(to) of how am confronting me to my illness……………………………………………… | | 0 | 1 | 2 | 3 | 4 |
| GE3 | I am losing the hopes in the fight against my  Illness ………………………………………........... | | 0 | 1 | 2 | 3 | 4 |
| GE4 | I seat me nervous(to) ..………………………………… | | 0 | 1 | 2 | 3 | 4 |
| GE5 | It concerns me die……………………………………… | | 0 | 1 | 2 | 3 | 4 |
| GE6 | It concerns me that my illness worsen……………... | | 0 | 1 | 2 | 3 | 4 |

|  | **CAPACITY OF PERSONAL OPERATION** | **At all** | | **A bit** | **Something** | **A lot** | **Muchí- simo** |
| --- | --- | --- | --- | --- | --- | --- | --- |
|  |  |  | |  |  |  |  |
| GF1 | I can work (it include the work in the home)…………. | | 0 | 1 | 2 | 3 | 4 |
| GF2 | My work satisfies me (include the work in the home)... | | 0 | 1 | 2 | 3 | 4 |
| GF3 | I can enjoy of the life………………………………. | | 0 | 1 | 2 | 3 | 4 |
| GF4 | I have accepted my illness…………………………….. | | 0 | 1 | 2 | 3 | 4 |
| GF5 | I sleep well…………………………………………….. | | 0 | 1 | 2 | 3 | 4 |
| GF6 | I enjoy with my hobbies of always……………… | | 0 | 1 | 2 | 3 | 4 |
| GF7 | I am satisfied(to) with my quality of current life……… | | 0 | 1 | 2 | 3 | 4 |

PUNCTUATION: ____+____+____+___

SESSION OF RELAXATION data session

1. Degree of previous anxiety to the session

0---1---2---3---4---5---6---7---8---9---10

1. Degree of relaxation reached

0---1---2---3---4---5---6---7---8---9---10

1. Degree of concentration in the exercises

0---1---2---3---4---5---6---7---8---9---10

1. Command of the technician

0---1---2---3---4---5---6---7---8---9---10

1. Confidence in his utility

0---1---2---3---4---5---6---7---8---9---10

*Being *0* at all and *10* the possible maximum.

Observations that the researcher consider notable:

TELEPHONE FOLLOW-UP DATA POST-SESSION

WEEK 1

1. It has made the technician in his domicile? In how many occasions? (Times/day)
2. Degree of previous anxiety to the session

0---1---2---3---4---5---6---7---8---9---10

1. Degree of relaxation reached

0---1---2---3---4---5---6---7---8---9---10

1. Degree of concentration in the exercises

0---1---2---3---4---5---6---7---8---9---10

1. Command of the technician

0---1---2---3---4---5---6---7---8---9---10

1. Confidence in his utility

0---1---2---3---4---5---6---7---8---9---10

1. It considers that the technical is helping him to control the symptoms derived of the anxiety (sleeplessness, nervousness, anxiety, muscular tension…)?

Which? How much in each case?

0---1---2---3---4---5---6---7---8---9---10 ­­­­­­­­­­­­­­­­­­­­­­­­­­­______________________

0---1---2---3---4---5---6---7---8---9---10 ______________________

0---1---2---3---4---5---6---7---8---9---10 ­­­­­­­­­­­­­­­­­­­­­­­­­­­______________________

0---1---2---3---4---5---6---7---8---9---10 ______________________

1. It takes some drug of hypnotic rescue or ansiolítico? IF NO

It has reduced the consumption? IF NO Which? How much?

1. Has pain? IF NO Scale EVA:

It considers that this technical helps him to control the pain? IF NO

-If it is that if, it Has reduced the consumption of analgesics? IF NO it does not take

-If it is that if, Which? How much? (Scale EVA)

1. Has nauseas? IF NO

It considers that this technical helps him to control the nauseas? IF NO

-If it is that if, it Has reduced the consumption of antieméticos? IF NO it does not take

-If it is that if, Which? How much?

Observations

WEEK 2

1. It has made the technician in his domicile? In how many occasions? (Times/day)

2. Degree of previous anxiety to the session

0---1---2---3---4---5---6---7---8---9---10

3. Degree of relaxation reached

0---1---2---3---4---5---6---7---8---9---10

4. Degree of concentration in the exercises

0---1---2---3---4---5---6---7---8---9---10

5. Command of the technician

0---1---2---3---4---5---6---7---8---9---10

6. Confidence in his utility

0---1---2---3---4---5---6---7---8---9---10

7. It considers that the technical is helping him to control the symptoms derived of the anxiety (sleeplessness, nervousness, anxiety, muscular tension…)?

Which? How much in each case?

0---1---2---3---4---5---6---7---8---9---10 ______________________

0---1---2---3---4---5---6---7---8---9---10 ______________________

0---1---2---3---4---5---6---7---8---9---10 ______________________

0---1---2---3---4---5---6---7---8---9---10 ______________________

8. It takes some drug of hypnotic rescue or ansiolítico? IF NO

It has reduced the consumption? IF NO Which? How much?

9. Has pain? IF NO Scale EVA:

It considers that this technical helps him to control the pain? IF NO

-If it is that if, it Has reduced the consumption of analgesics? IF NO it does not take

-If it is that if, Which? How much? (Scale EVA)

10. Has nauseas? IF NO

It considers that this technical helps him to control the nauseas? IF NO

-If it is that if, it Has reduced the consumption of antieméticos? IF NO it does not take

-If it is that if, Which? How much?

Observations

WEEK 3

1. It has made the technician in his domicile? In how many occasions? (Times/day)

2. Degree of previous anxiety to the session

0---1---2---3---4---5---6---7---8---9---10

3. Degree of relaxation reached

0---1---2---3---4---5---6---7---8---9---10

4. Degree of concentration in the exercises

0---1---2---3---4---5---6---7---8---9---10

5. Command of the technician

0---1---2---3---4---5---6---7---8---9---10

6. Confidence in his utility

0---1---2---3---4---5---6---7---8---9---10

7. It considers that the technical is helping him to control the symptoms derived of the anxiety (sleeplessness, nervousness, anxiety, muscular tension…)?

Which? How much in each case?

0---1---2---3---4---5---6---7---8---9---10 ______________________

0---1---2---3---4---5---6---7---8---9---10 ______________________

0---1---2---3---4---5---6---7---8---9---10 ______________________

0---1---2---3---4---5---6---7---8---9---10 ______________________

8. It takes some drug of hypnotic rescue or ansiolítico? IF NO

It has reduced the consumption? IF NO Which? How much?

9. Has pain? IF NO Scale EVA:

It considers that this technical helps him to control the pain? IF NO

-If it is that if, it Has reduced the consumption of analgesics? IF NO it does not take

-If it is that if, Which? How much? (Scale EVA)

10. Has nauseas? IF NO

It considers that this technical helps him to control the nauseas? IF NO

-If it is that if, it Has reduced the consumption of antieméticos? IF NO it does not take

-If it is that if, Which? How much?

Observations

WEEK 4

1. It has made the technician in his domicile? In how many occasions? (Times/day)

2. Degree of previous anxiety to the session

0---1---2---3---4---5---6---7---8---9---10

3. Degree of relaxation reached

0---1---2---3---4---5---6---7---8---9---10

4. Degree of concentration in the exercises

0---1---2---3---4---5---6---7---8---9---10

5. Command of the technician

0---1---2---3---4---5---6---7---8---9---10

6. Confidence in his utility

0---1---2---3---4---5---6---7---8---9---10

7. It considers that the technical is helping him to control the symptoms derived of the anxiety (sleeplessness, nervousness, anxiety, muscular tension…)?

Which? How much in each case?

0---1---2---3---4---5---6---7---8---9---10 ______________________

0---1---2---3---4---5---6---7---8---9---10 ______________________

0---1---2---3---4---5---6---7---8---9---10 ______________________

0---1---2---3---4---5---6---7---8---9---10 ______________________

8. It takes some drug of hypnotic rescue or ansiolítico? IF NO

It has reduced the consumption? IF NO Which? How much?

9. Has pain? IF NO Scale EVA:

It considers that this technical helps him to control the pain? IF NO

-If it is that if, it Has reduced the consumption of analgesics? IF NO it does not take

-If it is that if, Which? How much? (Scale EVA)

10. Has nauseas? IF NO

It considers that this technical helps him to control the nauseas? IF NO

-If it is that if, it Has reduced the consumption of antieméticos? IF NO it does not take

-If it is that if, Which? How much?

Observations

Quality of Life Scale Fact-G: final DATA POST-SESSION

Regarding the last 7 days:

|  | **GENERAL PHYSICAL STATE OF HEALTH** | **At all** | | **A bit** | **Something** | **A lot** | **Muchí- simo** |
| --- | --- | --- | --- | --- | --- | --- | --- |
|  |  |  | |  |  |  |  |
| GP1 | It is missing me energia................................................................ | 0 | | 1 | 2 | 3 | 4 |
| GP2 | Have nauseas.................................................................. | 0 | | 1 | 2 | 3 | 4 |
| GP3 | Because of my physical state, have difficulty to attend to the needs of my family……………..………….. | 0 | | 1 | 2 | 3 | 4 |
| GP4 | Have pain …………………………………………….. | 0 | | 1 | 2 | 3 | 4 |
| GP5 | They bother me the secondary effects of the treatment…... | 0 | | 1 | 2 | 3 | 4 |
| GP6 | I seat me ill(to)…………………………………… | 0 | | 1 | 2 | 3 | 4 |
| GP7 | I have to happen time put to bed(to)…………………… | 0 | | 1 | 2 | 3 | 4 |
|  | | | | | | | |
|  | **FAMILIAR And SOCIAL ENVIRONMENT** | **At all** | | **A bit** | **Something** | **A lot** | **Muchí- simo** |
|  |  |  | |  |  |  |  |
| GS1 | I seat me near(to) to my friendships.............................. | 0 | | 1 | 2 | 3 | 4 |
| GS2 | I receive emotional support by part of my family………. | 0 | | 1 | 2 | 3 | 4 |
| GS3 | I receive support by part of my friendships………………. | 0 | | 1 | 2 | 3 | 4 |
| GS4 | My family has accepted my illness………………… | 0 | | 1 | 2 | 3 | 4 |
| GS5 | I am satisfied(to) with the way in that it communicates my family about my illness…………………….  0 | 0 | | 1 | 2 | 3 | 4 |
| GS6 | I seat me near(to) to my couple (or to the person that is my main source of support)…………………………...  0 | 0 | | 1 | 2 | 3 | 4 |
| Q1  GS7 | *Without mattering his current level of sexual activity, answer to the following question. If it prefers to not to answer it, it mark this box*  *And it continue with the following section.* | | |  |  |  |  |
| GS7 | I am satisfied(to) with my sexual life………………… | | 0 | 1 | 2 | 3 | 4 |

|  | **EMOTIONAL STATE** | **At all** | | **A bit** | **Something** | **A lot** | **Muchí- simo** |
| --- | --- | --- | --- | --- | --- | --- | --- |
|  |  |  | |  |  |  |  |
| GE1 | I seat me sad................................................................. | | 0 | 1 | 2 | 3 | 4 |
| GE2 | I am satisfied(to) of how am confronting me to my illness……………………………………………… | | 0 | 1 | 2 | 3 | 4 |
| GE3 | I am losing the hopes in the fight against my  Illness ………………………………………........... | | 0 | 1 | 2 | 3 | 4 |
| GE4 | I seat me nervous(to) ..………………………………… | | 0 | 1 | 2 | 3 | 4 |
| GE5 | It concerns me die……………………………………… | | 0 | 1 | 2 | 3 | 4 |
| GE6 | It concerns me that my illness worsen……………... | | 0 | 1 | 2 | 3 | 4 |

|  | **CAPACITY OF PERSONAL OPERATION** | **At all** | | **A bit** | **Something** | **A lot** | **Muchí- simo** |
| --- | --- | --- | --- | --- | --- | --- | --- |
|  |  |  | |  |  |  |  |
| GF1 | I can work (it include the work in the home)…………. | | 0 | 1 | 2 | 3 | 4 |
| GF2 | My work satisfies me (include the work in the home)... | | 0 | 1 | 2 | 3 | 4 |
| GF3 | I can enjoy of the life………………………………. | | 0 | 1 | 2 | 3 | 4 |
| GF4 | I have accepted my illness…………………………….. | | 0 | 1 | 2 | 3 | 4 |
| GF5 | I sleep well…………………………………………….. | | 0 | 1 | 2 | 3 | 4 |
| GF6 | I enjoy with my hobbies of always……………… | | 0 | 1 | 2 | 3 | 4 |
| GF7 | I am satisfied(to) with my quality of current life……… | | 0 | 1 | 2 | 3 | 4 |

PUNCTUATION:____+____+____+____=____

**Annex 6. The Functional Assessment of Cancer Therapy-General.**

the functional assessment of cancer therapAnd general

To continuation will find a list of affirmations that other people with his same illness consider important. **It mark an alone number by line to indicate the answer that corresponds to the last 7 days.**

|  | **GENERAL PHYSICAL STATE OF HEALTH** | **At all** | | **A bit** | **Something** | **A lot** | **Muchí- simo** |
| --- | --- | --- | --- | --- | --- | --- | --- |
|  |  |  | |  |  |  |  |
| GP1 | It is missing me energy | 0 | | 1 | 2 | 3 | 4 |
| GP2 | Have nauseas | 0 | | 1 | 2 | 3 | 4 |
| GP3 | Because of my physical state, have difficulty to attend to the needs of my family. | 0 | | 1 | 2 | 3 | 4 |
| GP4 | Have pain | 0 | | 1 | 2 | 3 | 4 |
| GP5 | They bother me the efectyou secondary of the treatment | 0 | | 1 | 2 | 3 | 4 |
| GP6 | I seat me ill(to) | 0 | | 1 | 2 | 3 | 4 |
| GP7 | I have to happen time put to bed(to) 0 | 0 | | 1 | 2 | 3 | 4 |
|  | | | | | | | |
|  | **FAMILIAR And SOCIAL ENVIRONMENT** | **At all** | | **A bit** | **Something** | **A lot** | **Muchí- simo** |
|  |  |  | |  |  |  |  |
| GS1 | Me siento near(to) to my friendships | 0 | | 1 | 2 | 3 | 4 |
| GS2 | I receive support emocional by part of my family | 0 | | 1 | 2 | 3 | 4 |
| GS3 | Receipt apoyo by part of my friendships | 0 | | 1 | 2 | 3 | 4 |
| GS4 | My familia has accepted my illness | 0 | | 1 | 2 | 3 | 4 |
| GS5 | I am satisfied(to) with the way in that it communicates my family about my illness 0 | 0 | | 1 | 2 | 3 | 4 |
| GS6 | I seat me near(to) to my couple (or to the person that is my main source of support) 0 | 0 | | 1 | 2 | 3 | 4 |
| Q1  GS7 | *Without mattering his current level of sexual activity, answer to the following question. If it prefers to not to answer it, it mark this box*  *And it continue with the following section.* | | |  |  |  |  |
| GS7 | I am satisfied(to) with my sexual life | | 0 | 1 | 2 | 3 | 4 |

|  | **EMOTIONAL STATE** | **At all** | | **A bit** | **Something** | **A lot** | **Muchí- simo** |
| --- | --- | --- | --- | --- | --- | --- | --- |
|  |  |  | |  |  |  |  |
| GE1 | I seat me sad | | 0 | 1 | 2 | 3 | 4 |
| GE2 | I am satisfied(to) of how me estoy confronting to my illness | | 0 | 1 | 2 | 3 | 4 |
| GE3 | I am losing the hopes in the fight against my  Illness | | 0 | 1 | 2 | 3 | 4 |
| GE4 | I seat me nervous(to) | | 0 | 1 | 2 | 3 | 4 |
| GE5 | It concerns me die | | 0 | 1 | 2 | 3 | 4 |
| GE6 | It concerns me that my illness worsen 0 | | 0 | 1 | 2 | 3 | 4 |

|  | **CAPACITY OF PERSONAL OPERATION** | **At all** | | **A bit** | **Something** | **A lot** | **Muchí- simo** |
| --- | --- | --- | --- | --- | --- | --- | --- |
|  |  |  | |  |  |  |  |
| GF1 | I can work (incluya the work in the home) | | 0 | 1 | 2 | 3 | 4 |
| GF2 | My work satisfies me (incluya the work in the home) | | 0 | 1 | 2 | 3 | 4 |
| GF3 | I can enjoy of the life | | 0 | 1 | 2 | 3 | 4 |
| GF4 | I have accepted my illness | | 0 | 1 | 2 | 3 | 4 |
| GF5 | I sleep well | | 0 | 1 | 2 | 3 | 4 |
| GF6 | I enjoy with my hobbies of always | | 0 | 1 | 2 | 3 | 4 |
| GF7 | I am satisfied(to) with my quality of current life 0 | | 0 | 1 | 2 | 3 | 4 |

**Annex . Informative triptych for the patient.**

**Annex. Informative leaf for the patient and consent informed.**

INFORMATIVE LEAF FOR THE PATIENT

TITLE OF THE STUDY

Technician of Progressive Muscular Relaxation in the Care of the Anxiety and the Pain in the Patient Oncologic.

RESEARCHERS

Paula Parás Bravo, Paloma Saviours, María Cristina Alonso White, Mercedes Rodríguez Rodríguez, María Valdor Arriaran, David Gonzalez Bravo, Mª Jesus Ortega Solano, Mirian Villar of the Heras, Alfredo Domínguez Cruz, Ángel Herrera of the Order, Lourdes Boxes Santana, Héctor Nafría Soria, Ana García Rumí, Nuria Claramonte Pujol, Mª Paz Fernandez Ortega, José Manuel López Moreno, Sandra Cabrera Jaime, Carmen Suárez Sierra, Transito Cartwright Egido, Mª Elena González Jarraque, Aranzazu Moral Herranz, Ana María Palaces Romero, María Asunción Sanz Jiménez, María Portal Mañeru.

CENTRE

University hospital Marquis of Valdecilla, Santander, Cantabria/Hospital Sierrallana, Torrelavega, Cantabria/University Hospital of Fuenlabrada, Madrid/University Hospital of Getafe, Madrid/University Hospital Door of the Iron-Majadahonda, Madrid/Catalan Institute of Oncology Hospitalet de Llobregat, Barcelona/Catalan Institute of Oncology Badalona, Barcelona/University Hospital of Salamanca, Salamanca/Foundation Hospital Alcorcón, Hospitable/Complex Madrid of Navarra, Pamplona.

INTRODUCTION

We head to you to inform him on a study of investigation in which it invites him to participate. The study has been approved by the Ethical Committee of Clinical Investigation of Cantabria fulfilling with the valid rule.

Our intention is to provide him suitable and sufficient information so that it can evaluate and judge if it wants to or not participating in the study. For this read with attention this informative leaf and afterwards will be able to ask any doubt that arise him relative to the study. Besides it can consult with any person that consider timely.

Voluntary participation

It has to know that his participation in this study is voluntary and can decide not participating. In case that it decide to participate in the study can change his decision and withdraw his consent anytime, without that thus it alter the relation with the professionals that attend him and without that produce damage any in his treatment.

General description of the study

The aim of this study is to help to people that have difficulty to relax .

For this have designed a project, framed inside a thesis doctoral, whose length will be roughly two years and in which they go to participate the patients of the services of oncohematología of several hospitals of the National System of Health.

The study has two groups, one on which take part (group intervention), and to the that invite him to participate to you, and another that serves us of control and that creates through the clinical histories of the patients of the two last years (group control).

To the participants go them to teach a technician of relaxation and like this can help him to control symptoms incomodos as it can be the nervousness and the sleeplessness.

What want to study is if the technician of relaxation helps to reduce the symptoms derived of the anxiety and the pain.

For this will use the Technician of Relaxation of Jacobson. It treats of a safe technician that does not have hurtful effects for you and that does not interfere in his medical treatment.

To be able to learn it go him to quote and go to make the technician in groups reduced during roughly an hour. When you learn the technician will take very few minutes in making it.

It treats of a simple technician that makes seated following verbal indications. It does not require any previous physical skill.

These sessions will make them in the hospital in a place enabled to such effect.

The schedule is flexible by what can quote him the day and the hour that more suit him.

The session is directed by nurses with the necessary knowledges for this.

Later and during a month will call him once a week to know his evolution.

Also we will consult some data of his clinical history like the diagnostic, the treatment, etc. All the information will be treated of confidential form in accordance with the valid legislation.

During all the process can interrupt his participation if it wishes it without that this suppose an alteration in the cares that receives.

Of his participation in this project does not derive any cost for you neither any economic reward.

His participation is very important and appreciate it to him since no only goes to learn a technician of relaxation used internationally if no that with his generosity will be able to help to other people in his same situation.

Profits of the participation in the study

It expects improve the relative scientific knowledge to the disorders of anxiety and the pain of the patient with cancer and can that other patients benefit in the future. It is possible that you do not receive any direct profit in his health by his participation in this study.

Risks of the participation in the study

We will use the Technician of Relaxation of Jacobson. It treats of a safe technician that does not have hurtful effects for you and that does not interfere in his medical treatment.

CONFIDENTIALITY

All the data of personal character will treat of agreement to the had in the Organic Law 15/1999, of 13 December, of Data protection of Personal Character and the Royal decree 1720/2007, of 21 December, by which approves the Regulation that develops it.

The data collected for the study will be identified by means of a code so that it was not possible the identification of the patient. Only the researcher and people authorised related with the study will have access to said code and engage to use this information exclusively for the ends posed in the study. The members of the Ethical Committee of Clinical Investigation or Sanitary Authorities can have access to this information in fulfillment of legal requirements. It will preserve the confidentiality of these data and will not be able to be related with you, even although the results of the study are published.

DATA OF CONTACT

If has doubts anytime can contact with the responsible nurses of the study or to in this direction: Paula Parás Bravo Tfno.942200954 Email: [paula.paras@unican.es](mailto:paula.paras@unican.es)

CONSENT INFORMED

TITLE OF THE STUDY

Technician of Progressive Muscular Relaxation in the Care of the Anxiety and the Pain in the Patient Oncologic

RESEARCHERS

Paula Parás Bravo, Paloma Saviours, María Cristina Alonso White, Mercedes Rodríguez Rodríguez, María Valdor Arriaran, David Gonzalez Bravo, Mª Jesus Ortega Solano, Mirian Villar of the Heras, Alfredo Domínguez Cruz, Ángel Herrera of the Order, Lourdes Boxes Santana, Héctor Nafría Soria, Ana García Rumí, Nuria Claramonte Pujol, Mª Paz Fernandez Ortega, José Manuel López Moreno, Sandra Cabrera Jaime, Carmen Suárez Sierra, Transito Cartwright Egido, Mª Elena González Jarraque, Aranzazu Moral Herranz, Ana María Palaces Romero, María Asunción Sanz Jiménez, María Portal Mañeru.

CENTRE

University hospital Marquis of Valdecilla, Santander, Cantabria/Hospital Sierrallana, Torrelavega, Cantabria/University Hospital of Fuenlabrada, Madrid/University Hospital of Getafe, Madrid/University Hospital Door of the Iron-Majadahonda, Madrid/Catalan Institute of Oncology Hospitalet de Llobregat, Barcelona/Catalan Institute of Oncology Badalona, Barcelona/University Hospital of Salamanca, Salamanca/Foundation Hospital Alcorcón, Hospitable/Complex Madrid of Navarra, Pamplona.

D./Dña._______________________________________________________________________________

(Name and surnames of the patient in upper case)

I have read and comprised the leaf of information that has delivered me on the up indicated study. I have received sufficient information on the study. I have made all the questions that have required on the study. I have spoken with the D./Dña ______________________________________________ With the one who have clarificado the possible doubts. I comprise that my participation is voluntary. I comprise that I can withdraw me of the study: when it want to, without giving explanations and without that repercuta in my cares. I comprise that the personal information that contribute will be confidential and will not show to anybody without my consent. I comprise that my participation in the study involves to authorise to access to my clinical history and participate in a Protocol of Relaxation in groups reduced. And presto freely my compliance to participate in the study.

Signature of the researcher Signs of the patient

Date ______________________________________________________________

*(The date has to be filled of fist and letter by the patient)*

------------------------------------------------------------------------------------------------------------------------------

REVOCACIÓN OF THE CONSENT:

I, D./Dña. ___________________________________________________________________ I withdraw the consent awarded for my participation in the up quoted study.

Date and signature:
